# Detecting Genetic Isolation in Human Populations: A Study of European Language Minorities

**DOI:** 10.1371/journal.pone.0056371

**Published:** 2013-02-13

**Authors:** Marco Capocasa, Cinzia Battaggia, Paolo Anagnostou, Francesco Montinaro, Ilaria Boschi, Gianmarco Ferri, Milena Alù, Valentina Coia, Federica Crivellaro, Giovanni Destro Bisol

**Affiliations:** 1 Dipartimento di Biologia Ambientale, Università “La Sapienza”, Rome, Italy; 2 Dipartimento Biologia e Biotecnologie “Charles Darwin”, Università La Sapienza, Rome, Italy; 3 Istituto Italiano di Antropologia, Rome, Italy; 4 Facolta di Medicina, Istituto di Medicina Legale, Università Cattolica, Rome, Italy; 5 Dipartimento ad Attività Integrata di Laboratori, Anatomia Patologica, Medicina Legale, Struttura Complessa di Medicina Legale, Università di Modena e Reggio Emilia, Modena, Italy; 6 Dipartimento di Filosofia, Storia e Beni culturali, Universita degli Studi di Trento, Trento, Italy; 7 Division of Biological Anthropology, Leverhulme Centre for Human Evolutionary Studies, Cambridge, United Kingdom; University of Florence, Italy

## Abstract

The identification of isolation signatures is fundamental to better understand the genetic structure of human populations and to test the relations between cultural factors and genetic variation. However, with current approaches, it is not possible to distinguish between the consequences of long-term isolation and the effects of reduced sample size, selection and differential gene flow. To overcome these limitations, we have integrated the analysis of classical genetic diversity measures with a Bayesian method to estimate gene flow and have carried out simulations based on the coalescent. Combining these approaches, we first tested whether the relatively short history of cultural and geographical isolation of four “linguistic islands” of the Eastern Alps (Lessinia, Sauris, Sappada and Timau) had left detectable signatures in their genetic structure. We then compared our findings to previous studies of European population isolates. Finally, we explored the importance of demographic and cultural factors in shaping genetic diversity among the groups under study. A combination of small initial effective size and continued genetic isolation from surrounding populations seems to provide a coherent explanation for the diversity observed among Sauris, Sappada and Timau, which was found to be substantially greater than in other groups of European isolated populations. Simulations of micro-evolutionary scenarios indicate that ethnicity might have been important in increasing genetic diversity among these culturally related and spatially close populations.

## Introduction

Identifying signatures of genetic isolation is more challenging in humans than in most other animal species. In fact, the relatively young evolutionary age of *Homo sapiens* and the great number of opportunities human populations had to meet and admix have limited the overall impact of genetic isolation in many instances [1]. Therefore, genetic diversity at molecular level is smaller among humans than in other primates and large-bodied mammals, while there is a general consensus regarding the unsuitability of the concept of race for our species [2], [3]. Nonetheless, the identification of genetically isolated human groups remains fundamental for at least three reasons. Firstly, a thorough understanding of the genetic structure of human populations cannot be achieved without identifying groups which depart from common backgrounds or do not comply with defined spatial patterns of genetic variation. Secondly, genetic isolation in humans is often hypothesized to be associated with cultural diversity, which provides an opportunity to test the relations between cultural factors (e.g. language) and population genetic structure [4]. Finally, studies of human genetic isolates have proven to be extremely useful for mapping genes for rare monogenic disorders and are thought to be valuable for a better understanding of common genetic diseases [5], [6].

Unfortunately, our current knowledge of genetic isolation in human populations is incomplete. This depends not only on an inadequate sampling of candidate populations and insufficient coverage for important regions, but also on the difficulties in detecting unambiguous signatures of genetic isolation. In contrast to the methodological advancements achieved in the study of isolation in natural populations (e.g. [7], [8]), current approaches in human population genetics are based on the evaluation of within and among-group diversity levels (e.g. [9], [10], [11]), but it remains difficult to distinguish between the effects of reduced sample size, purifying selection and differential admixture and the consequences of long-term isolation. More recent methods based on linkage disequilibrium may be used only for biparental markers [12], but their sensitivity to genetic isolation has been questioned [13].

The above-mentioned limitations are even more evident when using unilinearly transmitted polymorphisms, due to the fact that they behave as single loci in evolutionary terms. Nevertheless, these genetic systems continue to represent today an important tool to study geographically and/or culturally isolated populations. In fact, differently from most autosomal loci, there is a relative abundance of data for comparison, both for cosmopolitan and admixed or small and remote groups. Furthermore, they are cheaper than panels of autosomal SNPs and less affected by ascertainment bias. It is also worth noting that unilinear markers provide a potential data basis for the application of some methods which are now being increasingly used in human population genetics [14], [15]. Examples include those based on Bayesian principles or developed from the coalescent algorithm, but that have yet to be adequately tested as tools for the study of human genetic isolation. On the whole, unilinear markers may help identify case studies of particular significance which could be further explored with more powerful approaches.

The present study aims to test whether a short history of cultural and geographical isolation may have left detectable genetic signatures in some European populations and, in a wider perspective, to assess the importance of demographic history and cultural factors in shaping genetic diversity across linguistic and/or geographic isolates on a continental scale. In order to overcome the limits of current approaches in detecting genetic isolation in human populations, we integrated classical genetic diversity measures with estimates of gene flow under an isolation with migration model. Combining these approaches, we first analyzed the genetic variation of mitochondrial DNA (mtDNA) polymorphisms in four German-speaking linguistic isolates from the Eastern Italian Alps (Sappada, Sauris, Timau and Lessinia). In order to put our results into a broader context, we built a large dataset which comprises both geographical and/or linguistic isolates and open populations from different parts of the European continent. In this way, we were able to detect converging signatures of genetic isolation in three of the groups under study, Sappada, Sauris and Timau. We then extended our study to the investigation of Y chromosome polymorphisms and we used coalescent simulations in order to explore the role of effective size and gene flow in determining the diversity observed among cultural and geographical isolates from the Italian Alps.

## Materials and Methods

### The population dataset

**Figure 1:**
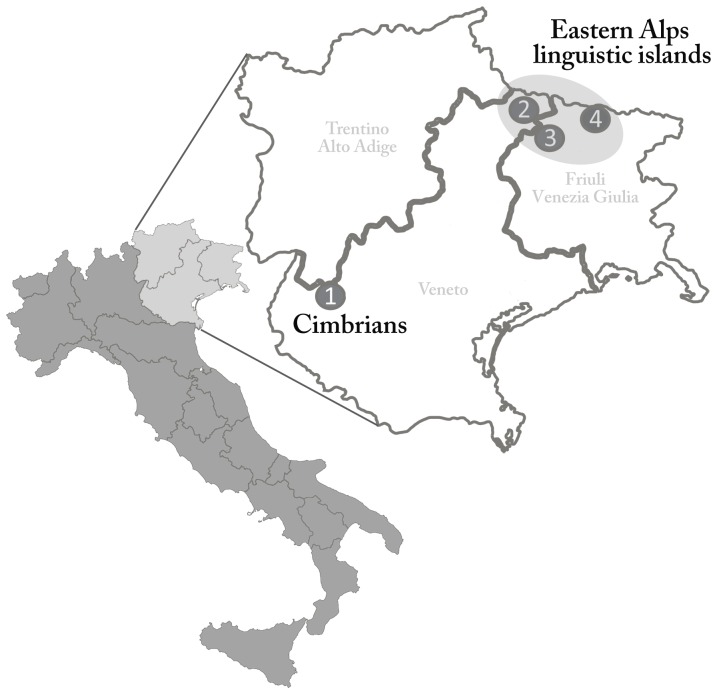
Geographic location of the populations analysed in this study. Population labels: (1) Lessinia; (2) Sappada; (3) Sauris; (4) Timau.

Our overall dataset comprises both unstudied populations and groups which have been analyzed in the course of previous research. The former include three linguistic islands of the Eastern Italian Alps (Sappada, Sauris and Timau) and a Cimbrian group from the Eastern pre-Alps (Lessinia) (Figure 1). Sappada (46°34′N 12°41′E) is a municipality of 1307 inhabitants [16] located at an altitude of 1245 m.a.s.l. on the North-Eastern Dolomite Alps in the province of Belluno in the Veneto region. The first settlers from Carinthia and Tyrol are thought to have arrived in the eleventh century AD [17]. Sauris and Timau are two villages of the Carnic Alps in the province of Udine in the Friuli Venezia Giulia region. The former (46°28′5″N 12°41′3″E) has 429 inhabitants [16], is located in the upper Lumiei valley (1212 m.a.s.l.) and its founders probably came from the lower Carinthia and Austrian Tyrol in the thirteenth century AD [18]. Timau (46°32′0″N 13°1′0″E) is a small village of about 500 inhabitants, situated at 830 m.a.s.l. in the But valley. The foundation of the community is traditionally said to have arisen from two different migration events from the neighboring Austrian region of Carinthia in the eleventh and thirteenth century AD [19]. The first Cimbrian settlers probably came from Bavaria around the eleventh century AD and settled in the nearby mountainous areas of Asiago, Luserna/Lavarone and Lessinia [20]. This latter area, which boasts a population of 13,455 inhabitants, is a mountainous territory in the province of Verona in the Veneto region on the border with Trentino [16]. The samples were collected in Giazza (45°39′11″N 11°7′21″E).

Despite a certain degree of cultural exchange with the surrounding neo-latin groups, these ethno-linguistic isolates have maintained a common cultural background and traditions [17], [19], [21], [22]. The dialects spoken in Sappada, Sauris and Timau have maintained a common south Bavarian background, with minor differences due to influences of Tyrolean dialects in Sappada and Sauris and Carinthian dialects in Timau. The Cimbrian language of Lessinia is an old western Tyrolean dialect and is currently spoken by a few dozen people in the community [23], [24].

Data produced in the course of this study were combined with results available in literature and online databases [25]. A first dataset consists of sequences of the hypervariable (HVR) regions 1 (from np 16033 to 16365) and 2 (np 073 to 340) from a total of 20 European populations (see Table S1). In order to increase the number of comparisons among populations, we built a second and larger mtDNA database (46 populations and 4198 individuals; see Table S2) of HVR-1 sequences only.

### Ethics statement

The research project was approved by the institutional review board of the *Istituto Italiano di Antropologia*. An appropriate informed consent with a withdrawal option was signed by all donors, and all their data were anonymized according to the “Decreto Legislativo della Repubblica Italiana, n° 196/2003”.

### Laboratory analyses

Buccal swabs were collected from a total of 193 individuals, comprising a sample of 40 from Lessinia, 59 from Sappada, 48 from Sauris and 46 from Timau. Donors were selected only if they were unrelated to other donors at grandparent level and with known-family origin. DNA was extracted using a modified “salting-out” procedure and HVR-1 and HVR-2 were amplified by PCR (primers: L-15990 and H-16501 for HVR-1; L-029 and H-408 for HVR-2). Amplified DNA was purified using a High Pure PCR Product Purification Kit (Roche Diagnostics, Mannheim, Germany), sequenced and compared with the Cambridge Reference Sequence rCRS [26]. Seventeen single-nucleotide polymorphisms (SNPs) of the mtDNA coding region (3010, 3915, 3992, 4216, 4336, 4529, 4580, 4769, 4793, 6776, 7028, 10398, 10400, 10873, 12308, 12705, 14766) were typed as reported in Quintans et al. 2004 [27]. Haplogroups were assigned according to Phylotree (version 14; [28]).

### Intra- and interpopulation genetic variation analysis

Haplotype diversity (HD) and its standard error were calculated according to Nei 1987 [29]. Pairwise differences among all the populations of the datasets were calculated using the genetic distance measure Fst [30], [31]. Analyses of molecular variance (AMOVA) were performed in order to examine genetic differences among populations of the same ethnic group [32]. Demographic descriptive indexes (Fu's Fs and Harpending's raggedness) were calculated to check for signs of demographic expansion [33], [34]. All the above parameters were calculated using Arlequin 3.5 [35]. Multidimensional scaling (MDS) was applied to genetic distance matrices to visualize genetic differentiation among populations using the SPSS software (release 16.0.1 for Windows, S.P.S.S. Inc.).

### Gene flow estimates

The IMa2 software, which applies the Isolation with Migration model, was used to estimate gene flow between populations [36], [37]. We considered population pairs formed by each of the surveyed linguistic isolates and a neighbouring population without a known history of geographical or cultural isolation factors (Cadore for Sappada and Udine for both Sauris and Timau) and a wide European population. The latter was obtained by pooling 7 open populations (Central Italy, France, North-East Germany, West Germany, Portugal, Spain, West Austria) whose pairwise Fst were found to be statistically insignificant. Since carrying out IMa2 runs with the entire pool of European populations (a total of 1137 individuals) was computationally too demanding, we used a subsample chosen comparing 100 subsamples of different size (50,100,150 and 200) to the entire dataset. The ones with n = 100 were found to provide the best combination of reduced computational times and substantial similarity to the original dataset, as evaluated comparing the original and subsampled datasets for HD, Fst, Fu's Fs, Tajima's D, Harpending's Raggedness and θ_H_ [29], [30], [31], [33], [34], [38], [39].

In order to allow comparisons among gene flow estimates, IMa2 runs were performed with priors which were kept constant for all population pairs. Uniform priors were used for the estimation of effective population size (q = 0–6000) and splitting time (t = 0–2.7), whereas an exponential prior (mean = 0.2) for gene flow (m) was adopted (see IMa2 manual for parameter unit conversion; http://genfaculty.rutgers.edu/hey/software#IMa2). We performed 2*10^6^ MCMC steps with burn-in period of 10^6^, geometric heating (ha = 0.9; hb = 0.3) and 80 Metropolis-coupled chains. mtDNA sequences were assumed to mutate under the Hasegawa-Kishino-Yano (HKY) mutation model [40], with an overall substitution rate per year (μ = 5.2023*10^−5^ ) calculated according to the rates reported in Soares et al., 2009 [41]. For each pairwise population comparison, three independent runs with the same parameter settings, but different random number seeds, were performed. Convergence on the stationary distribution was considered to be reached when the independent runs provided similar unimodal posterior distributions for all the parameters (see Figure S1) and when the following conditions were verified for all runs: comparable estimated posterior density functions for the first (SET1) and second (SET2) half of the sampled genealogies, no long-term trends in L[P] and t plots, low autocorrelation values and an effective sample size that was higher than 50 for the t parameter. The average modal value obtained for each independent run was used as a parameter estimate. A detailed description of the results obtained is reported in the supplementary material (Table S4 and S5).

### Simulations

We generated random genealogies for three evolutionary scenarios with different effective population sizes and gene flow rates using the Fastsimcoal software [42]. These scenarios share a common evolutionary topology (figure S2) where three populations split from a large source population (effective population size = 10^5^; growth rate = 0.03) and then slowly expand (growth rate = 0.017). We used a uniform distribution for splitting times (32–48 generations), with an unequal gene flow between source and sink populations (0.0001 from source to sink and 0.001 in the opposite direction). The three scenarios for mtDNA were set as follows (with all prior distributions set as uniform): 1) Sink population effective size = 100–300, gene flow between sink populations = 0–0.005; 2) Sink population effective size = 100–300, gene flow between sink populations = 0.015–0.02; 3). Sink population effective size = 700–900, gene flow between sink populations = 0–0.005. For Y chromosome, we used the same values of effective size but halved gene flow in order to account for the effects of patrilocality in the model. We simulated 10^4^ genealogies for each scenario for both mtDNA (333 bp) and Y chromosome (5 STRs) using mutation-rate estimates for HVR-1 by Soares et al., 2009 [41] and DYS19, 390, 391, 392 and 393 by Ballantyne et al., 2010 [43] and assuming a generation time of 25 years. We randomly sampled 50 individuals from each sink population and analyzed their within-group diversity for each simulation using Arlequin 3.5 [35].

## Results

### Mitochondrial variation in the North-Eastern Italian Alps

A total of 87 different haplotypes were observed in the four populations sampled using HVR-1, HVR-2 and 17 SNPs. They were first assigned to 12 main haplogroups (H, HV, I, J, K, N, R, T, U, V, W, X) and, then, further classified into 48 sub-haplogroups (see Table S3) according to the updated phylogenetic tree of global human mitochondrial DNA variation (Phylotree Build 14). The most common haplogroups were found to be H for Lessinia (60%) and Timau (36.9%), U for Sauris (35.4%) and K for Sappada (44.1%). The latter represents the most evident departure from the haplogroup frequencies observed in European populations, where K is found at frequencies that range between 2% and 12% [44].

Comparing our results (Table 1) to available HVR-1 and HVR-2 literature data for European populations (Table S1), it is evident that three out of the four groups investigated are characterized by a reduced intra-population genetic variability. In fact, HD values for Sappada (0.897±0.022), Sauris (0.928±0.021) and Timau (0.936±0.017) are lower than most populations in the dataset, even when comparing range estimates incorporating 95% confidence intervals. By contrast, the HD value of Lessinia is not far from the figure reported for other European populations.

**Table 1:** Genetic diversity and demographic parameter estimates (HVR-1 and HVR-2) in the populations under study.

Population	Acr	N	K	HD (sd)	Fu's Fs (p-value)	r
Lessinia	LES	40	27	0.970 (0.013)	−14.549 (0.000)	0.011
Sappada	SAP	59	19	0.897 (0.022)	−1.320 (0.376)	0.049
Sauris	SAU	48	21	0.928 (0.021)	−2.540 (0.204)	0.012
Timau	TIM	46	20	0.936 (0.017)	−2.900 (0.166)	0.019

Abbreviations: Acr, acronym; n, sample size; k, number of haplotypes; HD, haplotype diversity, r, Harpending's raggedness.

**Figure 2:**
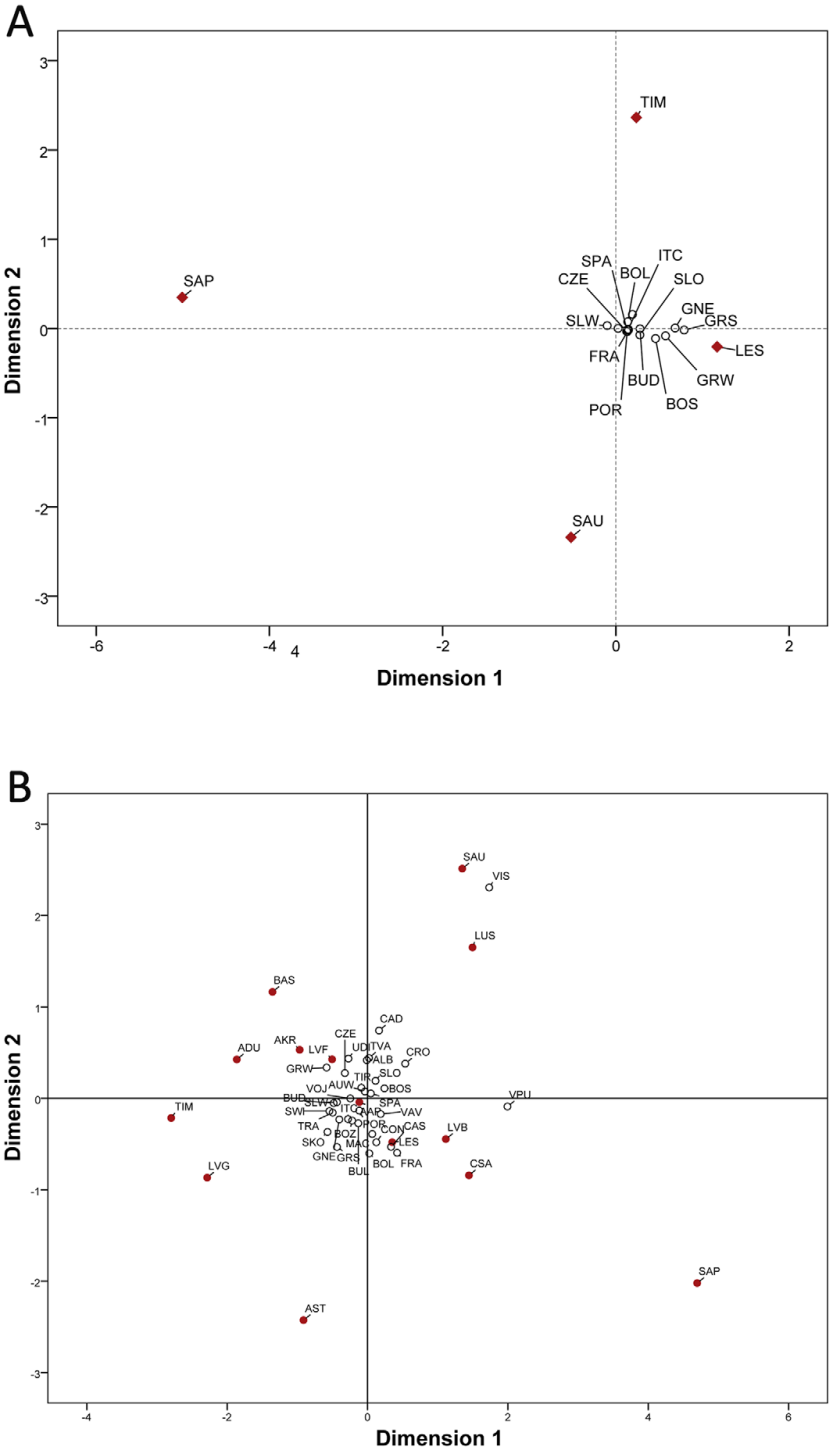
Multi-dimensional scaling plots of Fst genetic distances among European populations. (a) Plot based on mtDNA HVR1 and HVR2 sequences (stress value = 0.067). Population acronyms: BOL, Bologna; BOS, Bosnia; BUD, Budapest; ITC, Central Italy; CZE, Czech Republic; FRA, France; LES, Lessinia; GNE, North-East Germany; POR, Portugal; SAP, Sappada; SAU, Sauris; SLO, Slovenia; GRS, South Germany; SWI, South-West Switzerland; SPA, Spain; TIM, Timau; VOJ, Vojvodina; AUW, West Austria; GRW, West Germany; SLW, West Slovakia (References in Table S1); (b) Plot based on mtDNA HVR1 sequences only (stress value = 0.173). Populations acronyms as in Table S2. Linguistic and/or geographic isolates are marked in red.

The multi-dimensional scaling plot based on Fst values for both hypervariable regions (see Figure 2a) highlights the differentiation of Sappada, Sauris and Timau from other European populations, corroborated by the high statistical significance of all their genetic distances (p<0.01). As expected on the basis of the well known European genetic homogeneity, most populations cluster in the center of the plot. This group also includes Lessinia which shows an average genetic distance from the other populations which is 1.7–3.9 times lower than the other linguistic isolates (Table S1), with only 10 (out of 19) highly statistically significant pairwise values. We investigated the demographic history of the four studied populations using two different approaches. We obtained not-significant Fu's Fs values for Sappada, Sauris and Timau, which contrasts with Lessinia and all the other European populations analyzed. The lack of signatures of demographic expansion was further supported by mismatch distributions (Figure S3) and their raggedness values.

We replicated the analyses of intra- and inter-population genetic diversity using a dataset which was limited to HVR-1. However, the set contained a larger number of populations (46 vs 20 for the HVR-1/HVR-2 dataset), that included 14 European linguistic and/or geographic isolates. The reduced HD of Sappada, Sauris and Timau is reconfirmed (Table S2). Intriguingly, Sappada shows the lowest HD value even when compared to other language minorities which have been reported to be genetically isolated (Basques, Csángós, Ladins and Aromuns). The outlying position of Sappada, Sauris and Timau can also be observed in the MDS plot, and their divergence from other populations is greater than observed for other ethno-linguistic groups, such as Cimbrians, Ladins and Aromuns (Figure 2b; see Table S2). Even within a context of high inter-population differentiation, there is considerable diversity among the three groups, a fact shown by their marked reciprocal distance in the plot. Interestingly, some linguistic minorities which are not subject to strong geographic isolation (i.e. Basques from Spain, Csango from Romania and Aromuns Stip from Macedonia) gave a detectable signal of differentiation. This suggests there is a non-trivial association between linguistic and genetic diversity in our dataset.

### Estimating gene flow

As a more direct test of genetic isolation, we estimated incoming and outgoing gene flow between the populations that show robust signatures of isolation (Sappada, Sauris and Timau) and a neighbor or a Central Western European population. Due to the lack of HVR-2 sequences for neighbors, these analyses were performed using HVR-1 data only.

Table 2 displays the averaged values of three independent runs which converged on their marginal posterior probability distributions (see Table S4 for individual runs of gene flow, effective size and splitting time and Table S5 for mixing evaluation parameters). IMa2 seems to overestimate effective size and splitting time for linguistic isolates compared to our present demographic and historical knowledge [17], [18], [19], [20], [45]. However, it should be noted that the ratios of effective size estimated in linguistic isolates and neighbors (from 0.067 to 0.187) or the European reference population (from 0.016 to 0.063) is in line with their demographic history. An asymmetric gene flow between linguistic isolates and neighbors, with a 2∶1 ratio between outgoing and incoming, was observed. This imbalance becomes even more marked for Sappada and Sauris (ratios of 56∶1 and 155∶1, respectively) when replacing neighbors with a representative population of Central Western Europe. However, it must be said that confidence intervals overlap. While this may seem to indicate a non optimal power of the model for the estimate of individual parameters, an indication of the reliability of our inference is provided by the fact that confidence intervals for gene flow from open populations to linguistic isolates are more extended towards high values than vice versa, with a ratio between upper bound values that ranges from 10.3 (from Sappada to Cadore) to 94.7 (from Sappada to CW Europe).

**Table 2:** Ratios of effective population size and estimates of gene flow.

Population pair	N1/N2	m1-2	m2-1
Sappada – Cadore	0.067	0.71	0.35
		(0.05–30.59)	(0.07–2.96)
Sauris – Udine	0.187	0.82	0.43
		(0.06–71.36)	(0.02–5.31)
Timau – Udine	0.147	1.22	0.41
		(0.10–237.87)	(0.02–5.77)
Sappada – CW Europe	0.016	23.71	0.42
		(4.70–207.35)	(0.10–2.19)
Sauris – CW Europe	0.029	57.21	0.37
		(13.37–340.36)	(0.02–4.21)
Timau – CW Europe	0.063	1.61	0.57
		(0.24–206.89)	(0.03–6.11)

Abbreviations: N1/N2, effective population size ratio between population 1 and population 2; m1-2, effective number of haplotypes migrating from population 1 to population 2 per year; m2-1, effective number of haplotypes migrating from population 2 to population 1 per year. 95% credibility intervals in brackets.

### Analysis of the molecular variance

We further analysed the genetic diversity among populations carrying out an analysis of the molecular variance using both mtDNA and Y chromosome STRs (see Tables S1 and S6). We compared Eastern Alps linguistic islands and other European language minorities that show a comparable degree of cultural homogeneity and geographical proximity. These include Ladins and Cimbrians from the Eastern Alps and Aromuns from Albania and Macedonia (see Table 3).

**Table 3:** Analysis of Molecular Variance (AMOVA) in four groups of linguistic population isolates.

		mtDNA		Y chromosome	
Group	Populations	among pop.	p-value	among pop.	p-value
Eastern Alps Linguistic Islands	SAP-SAU-TIM	0.105	0.000	0.226	0.000
	SAP-TIM	0.136	0.000	0.187	0.000
	SAP-SAU	0.090	0.000	0.227	0.000
	SAU-TIM	0.090	0.000	0.261	0.000
Cimbrians	LES-LUS	0.023	0.073	-	-
Ladins	LVB-LVF-LVG	0.035	0.000	0.047	0.000
	LVB-LVG	0.055	0.001	0.031	0.026
	LVF-LVG	0.030	0.007	0.045	0.006
	LVB-LVF	0.020	0.002	0.062	0.008
Aromuns	AAP-ADU-AKR-AST	0.020	0.006	0.093	0.000
	AAP-ADU	0.006	0.253	0.204	0.000
	AKR-AST	0.024	0.043	0.040	0.005

Population acronyms and references: LES Lessinia, SAP Sappada, SAU Sauris and TIM Timau (This study); LVB Val Badia and LVG Val Gardena [10], LUS Luserna and LVF Val di Fassa ([66], Coia V. unpublished data); AAP Andon Poci, ADU Dukasi, AKR Krusevo and AST Stip [67].

Sappada, Sauris and Timau showed a value of among-population molecular variance which was three times higher for mtDNA and two times for Y chromosome. Interval estimates obtained for these populations (from 0.090 to 0.136) and other linguistic isolates (from 0.006 to 0.055) using a jackknife procedure do not overlap for mtDNA. Regarding Y chromosome, only the comparison between Albanian Aromuns from Dukasi and Andon Poci produced a value of among-group diversity (0.204) which is comparable to what we observed in German speaking linguistic islands from the Eastern Alps (from 0.187 to 0.261).

### Simulations of micro-evolutionary scenarios

**Figure 3:**
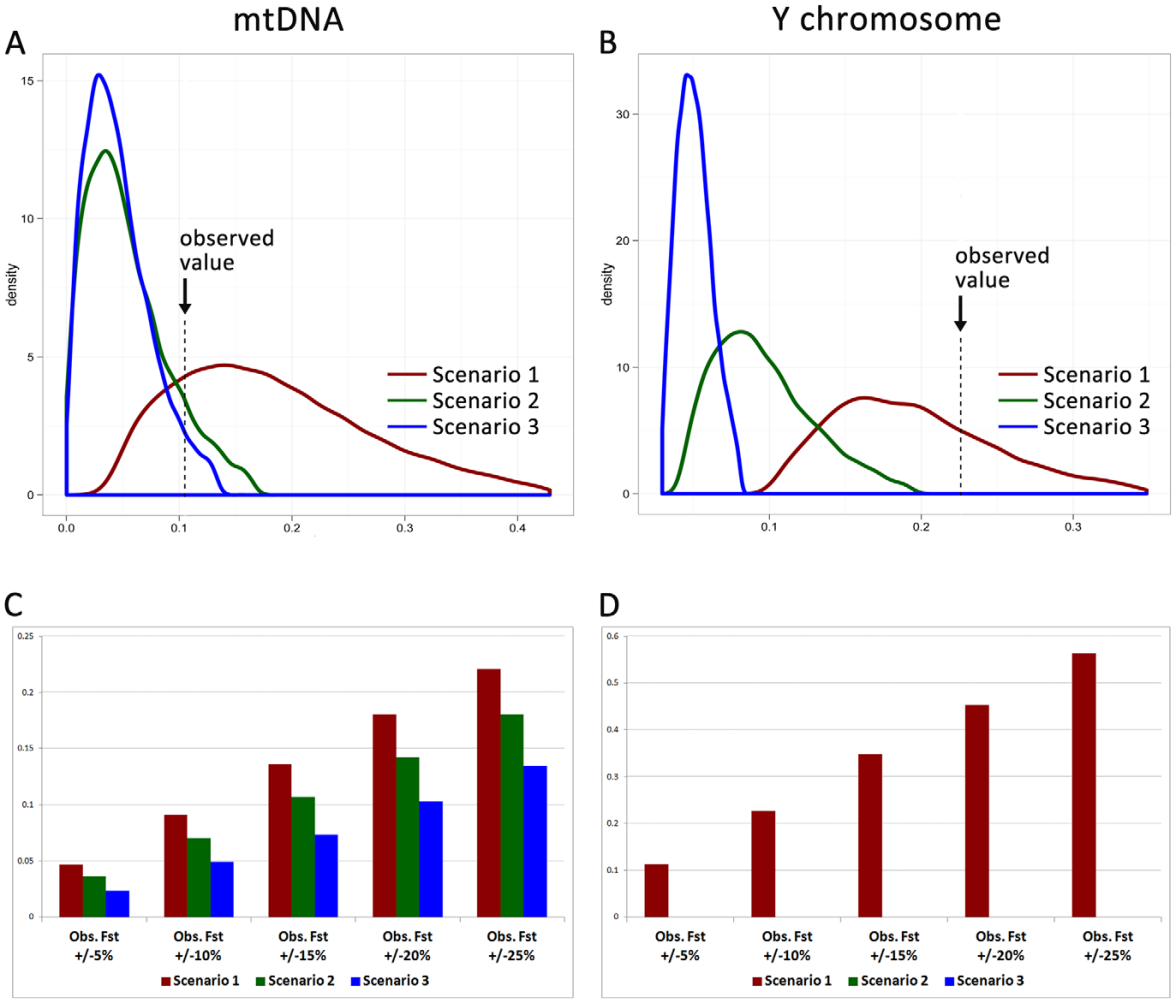
Posterior densities of Fst genetic distances for three micro-evolutionary scenarios. Frame A and B show mtDNA and Y chromosome diversity, respectively. Frames C and D show the proportion of mtDNA and Y chromosome simulations, respectively, with Fst values falling within different ranges around the observed Fst values (5%, 10%, 15%, 20%, and 25%, from left to right).

We first modeled a micro-evolutionary scenario for mtDNA and Y chromosome diversity in Sappada, Sauris and Timau fitting the historical knowledge regarding the splitting time and effective population size. As implied by the “local ethnicity” hypothesis (see below), we assumed an extremely low gene flow among populations. We, then, defined another two scenarios with varying degrees of gene flow and effective population size. Finally, we compared the 95% confidence intervals of distributions obtained for each scenario with observed Fst values (see figure 3).

The observed value of among population diversity (mtDNA, Fst = 0.105, p<0.0001; Y chromosome Fst = 0.226, p<0.0001) falls clearly within the range of the distributions expected under the “small effective size and low gene flow” scenario for both mtDNA and Y chromosome polymorphisms (Figure 3). Furthermore, all Y-chromosome and mtDNA Fst genetic distances in this model are statistically significant. To assess the relative importance of effective size and gene flow in the proposed scenario, we performed further simulations. As expected, increasing the effective size has a high impact on the genetic distances produced by simulations (see Figure S4 for further details). However, the results show that incrementing gene flow also led to substantially lower genetic distances for both genetic markers, which is not easy to predict given the small number of generations assumed in the simulations.

The other two hypotheses do not seem to be as well supported from simulations. Neither the “moderate effective size and low gene flow” nor the “small effective size and high gene flow” distributions of values encompass the observed Y chromosome Fst. For mtDNA, they are both compatible with the observations. However, the two alternative scenarios receive less support from the distribution of simulations that fall within different ranges of values around the observed Fst value (Figure 3), while less than 80% of genetic distances they produce are statistically significant.

## Discussion

### Detecting signatures of genetic isolation in the Alpine linguistic islands

The so called “Linguistic islands” of the Alps, small groups surrounded by communities that speak a distinct language [46], [47], provide a unique opportunity to study the combined effects of physical and cultural factors on human genetic diversity in a relatively small timescale. Having settled in their present day location in Medieval times, they can be regarded as “young isolates” according to the classification of Heutink & Oostra 2002 [48]. Within and among-group patterns of genetic variation observed for Sappada, Sauris and Timau, but not for Lessinia, are compatible with what is to be expected in “secondary isolates”, i.e. groups “derived from a relatively small population sample, which then slowly expand, with very little recruitment from outside the group” [49]. In fact, a significant HD reduction relative to open populations can be observed in the three groups, while they show a significant and high genetic distance from open European populations.

Interestingly, we were unable to detect any signatures of population expansion in Sappada, Sauris and Timau. However, this evidence is based on the analysis of gene pool of extant populations, so our results do not contradict a scenario in which the signatures of a population expansion could have been erased by a subsequent genetic drift event (see [50]). In our case, it may be hypothesized that the founder effect associated with the establishment of the new communities could have obliterated the genetic footprints of a previous expansion. Thereafter, their demographic growth and the number of generations elapsed since the founding event might not have been sufficient to restore signals of expansion.

However, as discussed in the introduction, all these results cannot be taken as definite proof of the presence of isolation. Further cause for caution comes from the fact that Sappada, Sauris and Timau have a small census size (from 429 to 1307). Unfortunately, there are no data for comparison from groups with a comparable demographic dimension, by which we could investigate the relations between census and population genetic measures when there is no genetic isolation.

For all the reasons discussed above, we decided to go one step further and apply a method for gene flow estimates based on Bayesian theory. This approach has been so far scantily adopted in human population genetics studies [51], [52], [53], and only one paper has focused on patterns of genetic isolation [54]. In our research, we made three methodological choices. Firstly, we used the IMa2 software because the model implemented therein (Isolation with Migration) fits the histories of populations which have experienced recent separation events (see “Introduction to the IM and IMa computer programs”, http://lifesci.rutgers.edu/%7Eheylab/ProgramsandData/Programs/IM/Introduction_to_IM_and_IMa_3_5_2007.pdf ). Secondly, we extended the analysis to a wide spectrum of populations, including neighbors and a reference European population. In this way, we were able to appreciate the different ratios between incoming and outgoing gene flow in populations with a different demographic history. Thirdly and finally, we adopted very stringent criteria for the validation of results (see Material and Methods) and kept priors constant throughout all IMa2 runs in order to guarantee a faithful comparison of results. As a side effect, gene flow estimates for some population pairs did not meet the standards established for results acceptance (see IMa2 manual, http://lifesci.rutgers.edu/%7Eheylab/ProgramsandData/Programs/IMa2/Using_IMa2_8_24_2011.pdf). In fact, priors set up for pairs formed by linguistic isolates and neighbors or reference population were found to be unfit for other population pairs, e.g. between isolates or between open populations. Even following these strict rules, however, we were able to detect coherent signatures of a substantially lower incoming gene flow in Sappada, Sauris and Timau compared to open neighboring groups. The difference was even more evident when the latter were replaced by a wide reference Central-Western European population. These results provide support to an unambiguous definition of Sappada, Sauris and Timau communities as genetic isolates, likely due to the combined effect of linguistic and geographical barriers to gene flow.

### Genetic diversity among related isolates: any role for “local ethnicity”?

There is a general consensus concerning the substantial homogeneity of the genetic structure of European populations relative to what can be observed in other continents [55], [56], [57]. However, looking at the distribution of human populations in greater detail, we can notice, especially in the Balkans and the Alps, the presence of numerous geographic and/or cultural isolates which could represent discontinuities in a relatively uniform genetic landscape. Some of these isolates originate from the subdivision of groups after an initial settlement or come from independent migrations from the same or nearby areas. The former case fits the ethnogenesis of Cimbrians, whereas the latter adapts to the establishment of linguistic islands of the Eastern Alps. Other dynamics which lead to the formation of isolates include the fragmentation and marginalization of populations that had previously settled in a wider area and which were later displaced by one or more massive migratory events. This scenario seems to fit the history of the Ladins from the Dolomites (Val Badia, Val Gardena and Val di Fassa) quite well [58], [59], [60].

All these processes have generated geographically-separated groups, even though they have remained often close to each other. While in most cases, they have maintained their original cultural traits, their level of genetic diversity remains to be established. To this purpose, we compared German speaking populations from the Eastern Alps with linguistic (Aromuns) and geo-linguistic isolates (Ladins, Cimbrians). The results of Amova show a greater within-group diversity for Y chromosome than for mtDNA, which is a likely effect of patrilocality. However, the main finding regards the high differentiation among Sappada, Sauris and Timau for both mtDNA and Y chromosome polymorphisms, both in absolute and comparative terms. How can we explain this result? The most obvious and likely reason could be that Sappada, Sauris and Timau were founded by small groups, as suggested by historical sources [17], [18], [19]. Since the three communities are relatively close each other (average distance 21 km vs 68 for Albanian Aromuns, 33 for Cimbrians, 13 for Ladins and 89 for Macedonian Aromuns), geographic distances do not seem to provide a simple explanation for their genetic differentiation. However, cultural factors might help us better understand the observed patterns. In fact, despite their close languages and shared traditions [61], [62], members of Alpine linguistic islands tend to identify their ancestry with their own village more than considering themselves as part of the same ethnic group [63], [64], [65]. By contrast, the sense of identity of Cimbrians, Ladins and Aromuns seems to be linked to the history and traditions of their common ethnic group rather than that of any single community or village.

Such a strong territoriality in defining ethnic identities and boundaries, which we name “local ethnicity”, may have played a role in marriage strategies, decreasing the genetic exchange among the three linguistic islands. Accordingly, a high level of endogamy has been observed in Sauris in biodemographic studies which cover a time period from the mid eighteenth to the mid nineteenth century [45], whereas no information is presently available for the other two communities.

To test this hypothesis, we used a heuristic approach based on coalescent simulations in a Bayesian framework. The high and statistically significant Fst values observed for Sappada, Sauris and Timau well fit the scenario modeled according to the “local ethnicity” hypothesis. Neither increasing the effective size nor assuming a higher gene flow, were we able to observe a comparable congruence between observed and simulated data. This suggests that a combination of small initial effective size with continued genetic isolation from surrounding populations and a reduced gene flow among communities may provide a worthwhile working hypothesis for the diversity observed among the linguistic islands of the Eastern Alps.

### Concluding Remarks

In this paper, we have attempted to overcome some of the limitations of current approaches regarding the study of genetic isolation in human populations using unilinear polymorphisms. Undoubtedly, there is room for further improvement. By increasing the resolution (e.g. sequencing the entire mtDNA molecule) or, even better, exploiting the greater potential of evolutionarily independent loci (i.e. autosomal SNPs) could help produce narrower estimates of gene flow and demographic parameters, and overcome the difficulties encountered when applying the IM method to populations with very different demographic histories. Similarly, our simulations could be seen as a first step towards the application of more complex and realistic scenarios. Even with these caveats, however, complementing classical measures of genetic diversity with Bayesian estimates of gene flow and simulations of micro-evolutionary models seems to be a suitable strategy to better understand genetic isolation and its relations with demographic and cultural factors in human populations.

## Supporting Information

File S1 Mitochondrial DNA and Y Chromosome raw data of the populations under study.
(XLS)

Table S1 mtDNA HVR-1 (16033–16365 np) and HVR-2 (73–340 np) genetic diversity and demographic parameter estimates in 20 European populations.
(DOC)

Table S2 mtDNA HVR-1 (16033–16365 np) genetic diversity and demographic parameter estimates in 46 European populations.
(DOC)

Table S3 Haplogroup frequency distribution in populations under study.
(DOC)

Table S4 Estimates of effective population size, gene flow and splitting time for all runs performed.
(DOC)

Table S5 Measures of MCMC mixing behavior.
(DOC)

Table S6 List of populations used for the analysis of Y chromosome STRs.
(DOC)

Figure S1 **Posterior distributions of parameter estimates.** Parameter abbreviations: q1, q2 and qa (effective population size of populations 1,2 and ancestral), T (splitting time), m1>2 (gene flow rate per haplotype from populations 2 to 1) and m2>1 (gene flow rate per haplotype from populations 1 to 2). A) Sappada vs Cadore; B) Sappada vs Central-Western Europe; C) Sauris vs Udine; D) Sauris vs Central-Western Europe; E) Timau vs Udine; F) Timau vs Central-Western Europe.
(PDF)

Figure S2 **Evolutionary topology used for the simulations of the three scenarios.**
(PDF)

Figure S3 **Nucleotide pairwise mismatch distribution for the populations under study.**
(PDF)

Figure S4 **Fst value distributions of simulated scenarios.** The gene flow distributions of the local ethnicity scenario with original (red line) and modified values (green lines) are shown in frames A and B whereas the effective size settings in frames C and D. Frames E and F show the percentual decrease of Fst modal values obtained with increasing values of gene flow and effective size.
(PDF)

## References

[1] Tishkoff SA, Kidd KK (2004) Implications of biogeography of human populations for ‘race’ and medicine. Nat Genet 36: S21–S27. 15507999 10.1038/ng1438

[2] Templeton AR (1998) Human races: a genetic and evolutionary perspective. Am Anthropol 100: 632–650.

[3] Barbujani G, Colonna V (2010) Human genome diversity: frequently asked questions. Trends Genet 26: 285–295. 20471132 10.1016/j.tig.2010.04.002

[4] Laland KN, Odling-Smee J, Myles S (2010) How culture shaped the human genome: bringing genetics and the human sciences together. Nat Rev Genet 11: 137–148. 20084086 10.1038/nrg2734

[5] Varilo T, Peltonen L (2004) Isolates and their potential use on complex gene mapping efforts. Curr Opin Genet Dev 14: 316–323. 15172676 10.1016/j.gde.2004.04.008

[6] Kristiansson K, Naukkarinen J, Peltonen L (2008) Isolated populations and complex disease gene identification. Genome Biol 9: 109. 18771588 10.1186/gb-2008-9-8-109 PMC2575505

[7] Eckert CG, Samis KE, Lougheed SC (2008) Genetic variation across species' geographical ranges: the central–marginal hypothesis and beyond. Mol Ecol 17: 1170–1188. 18302683 10.1111/j.1365-294X.2007.03659.x

[8] Hellberg ME (2009) Gene flow and isolation among populations of marine animals. Annu Rev Ecol Evol S 40: 291–310.

[9] Nasidze I, Quinque D, Udina I, Kunizheva S, Stoneking M (2006) The Gagauz, a linguistic enclave, are not a genetic isolate. Ann Hum Genet 71: 379–389. 17147693 10.1111/j.1469-1809.2006.00330.x

[10] Thomas MG, Barnes I, Weale ME, Jones AL, Foster P, et al (2008) New genetic evidence supports isolation and drift in the Ladin communities of the South Tyrolean alps but not an ancient origin in the Middle East. Eur J Hum Genet 16: 124–134. 17712356 10.1038/sj.ejhg.5201906

[11] van Oven M, Hämmerle JM, van Schoor M, Kushnick G, Pennekamp P, et al (2011) Unexpected island effects at an extreme: reduced Y chromosome and mitochondrial DNA diversity in Nias. Mol Biol Evol 28: 1349–1361. 21059792 10.1093/molbev/msq300

[12] Service S, DeYoung J, Karayiorgou M, Roos JL, Pretorious H, et al (2006) Magnitude and distribution of linkage disequilibrium in population isolates and implications for genome-wide association studies. Nat Genet 38: 556–560. 16582909 10.1038/ng1770

[13] Bosch E, Laayouni H, Morcillo-Suarez C, Casals F, Moreno-Estrada A, et al (2009) Decay of linkage disequilibrium within genes across HGDP-CEPH human samples: most population isolates do not show increased LD. BMC Genomics 10: 338. 19638193 10.1186/1471-2164-10-338 PMC2723139

[14] Tofanelli S, Taglioli L, Merlitti D, Paoli G (2011) Tools which simulate the evolution of uni-parentally transmitted elements of the human genome. J Anthropol Sci 89: 201–219. 21911915 10.4436/jass.89017

[15] Hoban S, Bertorelle G, Gaggiotti OE (2012) Computer simulations: tools for population and evolutionary genetics. Nat Rev Genet 13: 110–122. 22230817 10.1038/nrg3130

[16] ISTAT (2010) Bilancio Demografico e popolazione residente per sesso.

[17] Peratoner A (2002) Sappada/Plodn. Storia, etnografia e ambiente naturale. Pieve di Cadore: Tiziano Editore.

[18] Brunettin G (1998) L'insediamento di Sauris tra storiografia e rappresentazione di un'origine. In: Cozzi D, Isabella D, Navarra E, editors. Sauris, Zahre, una comunita' delle Alpi carniche. Udine: Forum Editrice Universitaria Udinese. pp. 43–61.

[19] Petris B (1980) Tischlbong Tamau Timau. Udine: Del Bianco.

[20] Rapelli G (2004) XIII comuni veronesi. La formazione dell'isola linguistica. In: Pezzi C, editor. Isole di cultura. Saggi sulle minoranze storiche germaniche in Italia. Luserna: Comitato Unitario delle Isole Linguistiche Storiche Germaniche in Italia-Centro Documentazione Luserna. pp. 243–248.

[21] Volpato G (1988) Fra tradizione popolare e antropologia storica. Per una comprensione del fenomeno “cimbro” dopo settecento anni. La Ricerca Folklorica 18: 117–123.

[22] Gri GP (1998) Zahre, Sauras, Sauris. In: Cozzi D, Isabella D, Navarra E, editors. Sauris Zahre, una comunità delle Alpi Carniche. Udine: Forum Editrice Universitaria Udinese. pp. 9–18.

[23] Maurer-Lausegger H (2004) The diversity of languages in the Alpine-Adriatic region I: linguistic minorities and enclaves in Northern Italy. Tidsskrift for Sprogforskning 2: 5–23.

[24] Toso F (2008) Le minoranze linguistiche in Italia. Bologna: il Mulino.

[25] Congiu A, Anagnostou P, Milia N, Capocasa M, Montinaro F, et al (2012) Online databases for mtDNA and Y chromosome polymorphisms in human populations. J Anthropol Sci 90: 197–212. 10.4436/jass.90020 23274751

[26] Andrews RM, Kubacka I, Chinnery PF, Lightowlers RN, Turnbull DM, et al (1999) Reanalysis and revision of the Cambridge reference sequence for human mitochondrial DNA. Nat Genet 23: 147. 10508508 10.1038/13779

[27] Quintáns B, Alvarez-Iglesias V, Salas A, Phillips C, Lareu MV, et al (2004) Typing of mitochondrial DNA coding region SNPs of forensic and anthropological interest using SNaPshot minisequencing. Forensic Sci Int 140: 251–257. 15036446 10.1016/j.forsciint.2003.12.005

[28] van Oven M, Kayser M (2009) Updated comprehensive phylogenetic tree of global human mitochondrial DNA variation. Hum Mutat 30: E386–E394. 18853457 10.1002/humu.20921

[29] Nei M (1987) Molecular Evolutionary Genetics. New York: Columbia University Press.

[30] Reynolds J, Weir BS, Cockerham CC (1983) Estimation for the coancestry coefficient: basis for a short-term genetic distance. Genetics 105: 767–779. 17246175 10.1093/genetics/105.3.767 PMC1202185

[31] Slatkin M (1995) A measure of population subdivision based on microsatellite allele frequencies. Genetics 139: 457–462. 7705646 10.1093/genetics/139.1.457 PMC1206343

[32] Excoffier L, Smouse P, Quattro J (1992) Analysis of molecular variance inferred from metric distances among DNA haplotypes: application to human mitochondrial DNA restriction data. Genetics 131: 479–491. 1644282 10.1093/genetics/131.2.479 PMC1205020

[33] Harpending RC (1994) Signature of ancient population growth in a low-resolution mitochondrial DNA mismatch distribution. Hum Biol 66: 591–600. 8088750

[34] Fu YX (1997) Statistical tests of neutrality of mutations against population growth, hitchhiking and backgroud selection. Genetics 147: 915–925. 9335623 10.1093/genetics/147.2.915 PMC1208208

[35] Excoffier L, Lischer HEL (2010) Arlequin suite ver 3.5: a new series of programs to perform population genetics analyses under Linux and Windows. Mol Ecol Resour 10: 564–567. 21565059 10.1111/j.1755-0998.2010.02847.x

[36] Nielsen R, Wakeley J (2001) Distinguishing migration from isolation. A Markov chain Monte Carlo approach. Genetics 158: 885–896. 11404349 10.1093/genetics/158.2.885 PMC1461674

[37] Hey J, Nielsen R (2007) Integration within the Felsenstein equation for improved Markov chain Monte Carlo methods in population genetics. P Natl Acad Sci Usa 104: 2785–2790. 10.1073/pnas.0611164104 PMC1815259 17301231

[38] Tajima F (1989) Statistical method for testing the neutral mutation hypothesis by DNA polymorphism. Genetics 123: 585–595. 2513255 10.1093/genetics/123.3.585 PMC1203831

[39] Chakraborty R, Weiss KM (1991) Genetic variation of the mitochondrial DNA genome in American Indians is at mutation-drift equilibrium. Am J Hum Genet 86: 497–506. 10.1002/ajpa.1330860405 1776656

[40] Hasegawa M, Kishino H, Yano T (1985) Dating of the human-ape splitting by a molecular clock of mitochondrial. DNA J Mol Evol 22: 160–174. 3934395 10.1007/BF02101694

[41] Soares P, Ermini L, Thomson N, Mormina M, Rito T, et al (2009) Correcting for purifying selection: an improved human mitochondrial molecular clock. Am J Hum Genet 84: 740–759. 19500773 10.1016/j.ajhg.2009.05.001 PMC2694979

[42] Excoffier L, Foll M (2011) Fastsimcoal: a continuous-time coalescent simulator of genomic diversity under arbitrarily complex evolutionary scenarios. Bioinformatics 27: 1332–1334. 21398675 10.1093/bioinformatics/btr124

[43] Ballantyne KN, Goedbloed M, Fang R, Schaap O, Lao O, et al (2010) Mutability of Y-chromosomal microsatellites: rates, characteristics, molecular bases, and forensic implications. Am J Hum Genet 87: 341–353. 20817138 10.1016/j.ajhg.2010.08.006 PMC2933352

[44] Ottoni C, Ricaut FX, Vanderheyden N, Brucato N, Waelkens M, et al (2011) Mitochondrial analysis of a Byzantine population reveals the differential impact of multiple historical events in South Anatolia. Eur J Hum Genet 19: 571–576. 21224890 10.1038/ejhg.2010.230 PMC3083616

[45] Navarra E (1998) Demografia di un villaggio alpino della Carnia: nuzialità e natalità a Sauris tra Settecento e Ottocento. La Ricerca Folklorica 38: 49–61.

[46] Viazzo PP (1989) Upland communities. Environment, population and social structure in the Alps since the sixteenth century. Cambridge: Cambridge University Press.

[47] Boattini A, Griso C, Pettener D (2011) Are ethnic minorities synonymous for genetic isolates? Comparing Walser and Romance populations in the Upper Lys Valley (Western Alps). J Anthropol Sci 89: 161–173. 21757790 10.4436/jass.89014

[48] Heutink P, Oostra BA (2002) Gene finding in genetically isolated populations. Hum Mol Genet 11: 2507–2515. 12351587 10.1093/hmg/11.20.2507

[49] Neel J (1992) Minority populations as genetic isolates: the interpretation of inbreeding results. In: Bittles AH, Roberts DF, editors. Minority Populations: Genetics Demography and Health. London: The MacMillan Press.

[50] Excoffier L, Schneider S (1999) Why hunter-gatherer populations do not show signs of pleistocene demographic expansions. Proc Natl Acad Sci U S A 96: 10597–10602. 10485871 10.1073/pnas.96.19.10597 PMC17928

[51] Garrigan D, Kingan SB, Pilkington MM, Wilder JA, Cox MP, et al (2007) Inferring human population sizes, divergence times and rates of gene flow from mitochondrial, X, and Y chromosome resequencing data. Genetics 177: 2195–2207. 18073427 10.1534/genetics.107.077495 PMC2219499

[52] Coelho M, Sequeira F, Luiselli D, Beleza S, Rocha J (2009) On the edge of Bantu expansions: mtDNA, Y chromosome and lactase persistence genetic variation in southwestern Angola. BMC Evol Biol 9: 80. 19383166 10.1186/1471-2148-9-80 PMC2682489

[53] Delfin F, Salvador JM, Calacal GC, Perdigon HB, Tabbada KA, et al (2011) The Y-chromosome landscape of the Philippines: extensive heterogeneity and varying genetic affinities of Negrito and non-Negrito groups. Eur J Hum Genet 19: 224–230. 20877414 10.1038/ejhg.2010.162 PMC3025791

[54] Brandstätter A, Egyed B, Zimmermann B, Duftner N, Padar Z, et al (2007) Migration rates and genetic structure of two Hungarian ethnic groups in Transylvania, Romania. Ann Hum Genet 71: 791–803. 17532745 10.1111/j.1469-1809.2007.00371.x

[55] Cavalli Sforza LL, Menozzi P, Piazza A (1993) The history and geography of human genes. Princeton: Princeton University Press.

[56] Torroni A, Achilli A, Macaulay V, Richards M, Bandelt HJ (2006) Harvesting the fruit of the human mtDNA tree. Trends Genet 22: 339–345. 16678300 10.1016/j.tig.2006.04.001

[57] Lao O, Lu TT, Nothnagel M, Junge O, Freitag-Wolf S, et al (2008) Correlation between genetic and geographic structure in Europe. Curr Biol 18: 1241–1248. 18691889 10.1016/j.cub.2008.07.049

[58] Pellegrini GB (1991) La genesi del Retoromanzo (o Ladino). Tübingen: Niemeyer.

[59] Loose R (1996) Siedlungsgeschichte des südlichen und mittleren Alpenraumes (Sudtirol, Trentino, Bellunese) seit der Karolingerzeit. Tiroler Heimat 60: 5–86.

[60] Kramer J (2004) La toponomastica altoatesina nel contesto europeo. Architettura Alto Adige 98: 277–290.

[61] De Concini W (1997) Gli altri delle Alpi. Minoranze linguistiche dell'arco alpino italiano. Trento: Comune di Pergine Valsugana.

[62] Navarra E (2002) Comportamenti demografici e organizzazione socio economica in due comunità germanofone delle Alpi orientali: Sappada e Sauris (sec. XVIII e XIX). In: Fornasin A Zannini A, editors. Uomini e comunità delle montagne. Udine: Forum Editrice Universitaria Udinese. pp. 113–132.

[63] Steinicke E (2001) Potential for conflicts in areas of ethno-linguistic minorities of the Eastern Alps. Annales 11: 259–266.

[64] Steinicke E, Piok E (2002) Le isole linguistiche di lingua tedesca a sud delle Alpi. Problematiche e conseguenze dell'identificazione etnica sull'esempio di Gressoney e di Timau. Tischlbongara Piachlan 6: 300–330.

[65] Steinicke E, Walder J, Löffler R, Beismann M (2011) Autochtonous linguistic minorities in the Italian Alps: new legislation – new identifications – new demographic processes. Journal of Alpine Research 99: 2.

[66] Coia V, Boschi I, Trombetta F, Cavulli F, Montinaro F, et al (2012) Evidence of high genetic variation among linguistically diverse populations on a micro-geographic scale: a case study of the Italian Alps. J Hum Genet 57: 254–260. 22418692 10.1038/jhg.2012.14

[67] Bosch E, Calafell F, González-Neira A, Flaiz C, Mateu E, et al (2006) Paternal and maternal lineages in the Balkans show a homogeneous landscape over linguistic barriers, except for the isolated Aromuns. Ann Hum Genet 70: 459–487. 16759179 10.1111/j.1469-1809.2005.00251.x

