# Supplementary material for: Detecting Genetic Isolation in Human Populations: A Study of European Language Minorities
Source: PLoS One. 2013 Feb 13;8(2):e56371. doi: 10.1371/journal.pone.0056371 (PMC3572090; doi:10.1371/journal.pone.0056371)
Supplement: Table S1 — mtDNA HVR-1 (16033–16365 np) and HVR-2 (73–340 np) genetic diversity and demographic parameter estimates in 20 European populations. (DOC) [file pone.0056371.s002.doc]

**Supplementary Table S1. mtDNA HVR-1 (16033-16365 np) and HVR-2 (73-340 np) genetic diversity and demographic parameter estimates in 20 European populations.**

| **Population** | **Acr** | **n** | **k** | **HD** | **HDsd** | **Fu's Fs** | **p Fs*** | **Avg. Fst** | **r** | **References** |
| --- | --- | --- | --- | --- | --- | --- | --- | --- | --- | --- |
| Bologna | BOL | 100 | 80 | 0.984 | 0.007 | -24.975 | 0.000 | 0.0117 | 0.005 | Bini et al. 2003 |
| Bosnia | BOS | 144 | 102 | 0.989 | 0.004 | -24.893 | 0.000 | 0.0114 | 0.005 | Malyarchuk et al. 2003 |
| Budapest | BUD | 211 | 171 | 0.992 | 0.003 | -24.569 | 0.001 | 0.0103 | 0.004 | Irwin et al. 2007 |
| Central Italy | ITC | 83 | 73 | 0.991 | 0.006 | -24.930 | 0.000 | 0.0085 | 0.005 | Tagliabracci et al. 2001 |
| Czech Republic | CZE | 93 | 78 | 0.991 | 0.005 | -24.728 | 0.000 | 0.0100 | 0.005 | Vanacek et al. 2004 |
| France | FRA | 50 | 46 | 0.996 | 0.005 | -25.148 | 0.000 | 0.0100 | 0.008 | Rousselet et al. 1998 |
| Lessinia (Cimbrians) | LES | 40 | 27 | 0.970 | 0.013 | -14.549 | 0.000 | 0.0224 | 0.011 | This study |
| North-East Germany | GNE | 300 | 204 | 0.993 | 0.002 | -24.502 | 0.001 | 0.0118 | 0.004 | Poetsch et al. 2003 |
| Portugal | POR | 241 | 179 | 0.987 | 0.004 | -24.464 | 0.000 | 0.0094 | 0.003 | Pereira et al. 2000 |
| Sappada | SAP | 59 | 19 | 0.897 | 0.022 | -1.320 | 0.376 | 0.0877 | 0.049 | This study |
| Sauris | SAU | 48 | 21 | 0.928 | 0.021 | -2.540 | 0.204 | 0.0416 | 0.012 | This study |
| Slovenia | SLO | 104 | 78 | 0.988 | 0.005 | -24.957 | 0.000 | 0.0102 | 0.004 | Malyarchuk et al. 2003 |
| South Germany | GRS | 200 | 140 | 0.982 | 0.005 | -24.787 | 0.000 | 0.0142 | 0.004 | Lutz et al. 1998 |
| South-West Switzerland | SWI | 154 | 123 | 0.994 | 0.002 | -24.674 | 0.000 | 0.0104 | 0.003 | Dimo-Simonin et al. 2000 |
| Spain | SPA | 312 | 227 | 0.995 | 0.001 | -24.349 | 0.000 | 0.0104 | 0.004 | Alvarez et al. 2007 |
| Timau | TIM | 46 | 20 | 0.936 | 0.017 | -2.900 | 0.166 | 0.0378 | 0.019 | This study |
| Vojvodina | VOJ | 104 | 87 | 0.995 | 0.002 | -24.797 | 0.000 | 0.0093 | 0.004 | Zgonjanin et al. 2010 |
| West Austria | AUW | 101 | 89 | 0.993 | 0.004 | -24.876 | 0.000 | 0.0084 | 0.004 | Parson et al. 1998 |
| West Germany | GRW | 50 | 43 | 0.989 | 0.008 | -25.141 | 0.000 | 0.0092 | 0.007 | Baasner et al. 1998 |
| West Slovakia | SLW | 70 | 64 | 0.997 | 0.003 | -24.770 | 0.000 | 0.0108 | 0.004 | Malyarchuk et al. 2008 |

Abbreviations: Acr, Acronym; n, Sample size; k, Number of haplotypes; HD, Haplotype Diversity; Avg Fst, Average Fst; r, Harpending’s Raggedness.

*significance at 5% level (p<0.02; see Fu 1997)

**References**

Alvarez JC, Johnson DLE, Lorente JA *et al.* (2007) Characterization of human control region sequences for Spanish individuals in a forensic mtDNA data set. *Legal Medicine*, **9**, 293-304.

Baasner A, Schäfer C, Junge A, Madea B (1998) Polymorphic sites in human mitochondrial DNA control region sequences: population data and maternal inheritance. *Forensic Science International*, **98**, 169-178.

Bini C, Ceccardi S, Luiselli D *et al.* (2003) Different informativeness of the three hypervariable mitochondrial DNA regions in the population of Bologna (Italy). *Forensic Science International*, **135**, 48-52.

Dimo-Simonin N, Grange F, Taroni F, Brandt-Casadevall C, Mangin P (2000) Forensic evaluation of mtDNA in a population from south west Switzerland. *International Journal of Legal Medicine*, **113**, 89-97.

Malyarchuck BA, Grzybowski T, Derenko MV *et al.* (2003) Mitochondrial DNA variability in Bosnians and Slovenians. *Annals of Human Genetics*, **67**, 412-425.

Malyarchuk BA, Perkova MA, Derenko MV *et al.* (2008) Mitochondrial DNA variability in Slovaks, with application to the Roma Origin. *Annals of Human Genetics*, **72**, 228-240.

Parson W, Parsons TJ, Scheithauer R, Holland MM (1998) Population data for 101 Austrian Caucasian mitochondrial DNA d-loop sequences: Application of mtDNA sequence analysis to a forensic case. *International Journal of Legal Medicine*, **111**, 124-132.

Pereira L, Prata MJ, Amorim A (2000). Diversity of mtDNA lineages in Portugal: not a genetic edge of European variation. *Annals of Human Genetics*, **64**, 491-506.

Poetsch M, Wittig H, Krause D, Lignitz E (2003) Mitochondrial diversity of a northeast German population sample. *Forensic Science International*, **137**, 125-132.

Rousselet F, Mangin P (1998) Mitochondrial DNA polymorphisms: a study of 50 French Caucasian individuals and application to forensic casework. *International Journal of Legal Medicine*, **111**, 292-298.

Tagliabracci A, Turchi C, Buscemi L, Sassaroli C (2001) Polymorphism of the mitochondrial DNA control region in Italians. *International Journal of Legal Medicine*, **114**, 224-228.

Vanacek T, Vorel F, Sip M (2004) Mitochondrial DNA D-loop hypervariable regions: Czech population data. *International Journal of Legal Medicine*, **118**, 14-18.

Zgonjanin D, Veselinović I, Kubat M *et al.* (2010) Sequence polymorphism of the mitochondrial DNA control region in the population of Vojvodina Province, Serbia. *Legal Medicine*, **12**, 104-107.
