# Supplementary material for: Detecting Genetic Isolation in Human Populations: A Study of European Language Minorities
Source: PLoS One. 2013 Feb 13;8(2):e56371. doi: 10.1371/journal.pone.0056371 (PMC3572090; doi:10.1371/journal.pone.0056371)
Supplement: Table S2 — mtDNA HVR-1 (16033–16365 np) genetic diversity and demographic parameter estimates in 46 European populations. (DOC) [file pone.0056371.s003.doc]

**Supplementary Table S2. mtDNA HVR-1 (16033-16365 np) genetic diversity and demographic parameter estimates in 46 European populations.**

| **Population** | **Acr** | **n** | **k** | **HD** | **HDsd** | **Fu's Fs** | **p Fs*** | | **Avg Fst** | **r** | **References** |
| --- | --- | --- | --- | --- | --- | --- | --- | --- | --- | --- | --- |
| Albania | ALB | 41 | 30 | 0.968 | 0.018 | -25.141 | 0.000 | 0.012 | | 0.015 | Belledi et al 2000 |
| Andon Poci (Aromuns) | AAP | 28 | 17 | 0.963 | 0.018 | -7.597 | 0.000 | 0.007 | | 0.050 | Bosch et al 2006 |
| Dukasi (Aromuns) | ADU | 33 | 19 | 0.964 | 0.015 | -9.789 | 0.000 | 0.027 | | 0.017 | Bosch et al 2006 |
| Krusevo (Aromuns) | AKR | 33 | 24 | 0.972 | 0.016 | -14.814 | 0.000 | 0.019 | | 0.027 | Bosch et al 2006 |
| Stip (Aromuns) | AST | 38 | 17 | 0.930 | 0.022 | -5.201 | 0.036 | 0.039 | | 0.019 | Bosch et al 2006 |
| Basques | BAS | 110 | 42 | 0.949 | 0.010 | -25.716 | 0.000 | 0.026 | | 0.020 | Cardoso et al 2011 |
| Bologna | BOL | 100 | 65 | 0.967 | 0.012 | -25.782 | 0.000 | 0.013 | | 0.010 | Bini et al 2003 |
| Bolzano | BOZ | 59 | 42 | 0.976 | 0.011 | -25.925 | 0.000 | 0.008 | | 0.015 | Thomas et al 2008 |
| Bosnia | BOS | 144 | 76 | 0.968 | 0.008 | -25.853 | 0.000 | 0.012 | | 0.015 | Malyarchuk et al 2003 |
| Budapest | BUD | 211 | 138 | 0.979 | 0.006 | -25.368 | 0.000 | 0.011 | | 0.013 | Irwin et al 2007 |
| Bulgaria | BUL | 110 | 72 | 0.978 | 0.007 | -25.737 | 0.000 | 0.011 | | 0.015 | Richards et al 2000 |
| Cadore | CAD | 32 | 24 | 0.976 | 0.015 | -15.534 | 0.000 | 0.015 | | 0.012 | UCSC unpublished data |
| Casentino | CAS | 122 | 78 | 0.979 | 0.007 | -25.662 | 0.000 | 0.012 | | 0.014 | Achilli et al 2007 |
| Central Italy | ITC | 83 | 62 | 0.974 | 0.012 | -25.655 | 0.000 | 0.009 | | 0.010 | Tagliabracci et al 2001 |
| Constanta | CON | 59 | 36 | 0.974 | 0.010 | -25.856 | 0.000 | 0.013 | | 0.021 | Bosch et al 2006 |
| Croatia | CRO | 96 | 48 | 0.975 | 0.006 | -26.064 | 0.000 | 0.018 | | 0.031 | Babalini et al 2005 |
| Csangos | CSA | 181 | 58 | 0.937 | 0.012 | -25.359 | 0.000 | 0.026 | | 0.029 | Brandstätter et al 2007 |
| Czech republic | CZE | 93 | 66 | 0.981 | 0.008 | -25.405 | 0.000 | 0.010 | | 0.008 | Vanacek et al 2004 |
| France | FRA | 50 | 42 | 0.988 | 0.009 | -25.810 | 0.000 | 0.010 | | 0.017 | Rousselet et al 1998 |
| Lessinia (Cimbrians) | LES | 40 | 23 | 0.953 | 0.017 | -13.817 | 0.000 | 0.018 | | 0.015 | This study |
| Luserna (Cimbrians) | LUS | 21 | 11 | 0.919 | 0.034 | -1.373 | 0.274 | 0.035 | | 0.073 | Coia et al 2012 |
| Macedonia | MAC | 182 | 94 | 0.975 | 0.006 | -25.465 | 0.000 | 0.010 | | 0.011 | Zimmermann et al 2007 |
| North-East Germany | GNE | 300 | 151 | 0.975 | 0.005 | -25.396 | 0.000 | 0.012 | | 0.014 | Poetsch et al 2003 |
| Portugal | POR | 241 | 144 | 0.959 | 0.010 | -25.254 | 0.000 | 0.010 | | 0.006 | Pereira et al 2000 |
| Sappada | SAP | 59 | 14 | 0.778 | 0.049 | -1.624 | 0.284 | 0.084 | | 0.134 | This study |
| Sauris | SAU | 48 | 19 | 0.923 | 0.020 | -4.293 | 0.070 | 0.045 | | 0.024 | This study |
| Skopje | SKO | 37 | 26 | 0.974 | 0.014 | -21.336 | 0.000 | 0.011 | | 0.034 | Bosch et al 2006 |
| Slovenia | SLO | 104 | 62 | 0.969 | 0.009 | -25.908 | 0.000 | 0.011 | | 0.016 | Malyarchuk et al 2003 |
| South Germany | GRS | 200 | 121 | 0.938 | 0.014 | -25.817 | 0.000 | 0.014 | | 0.011 | Lutz et al 1998 |
| South-West Switzerland | SWI | 154 | 97 | 0.976 | 0.007 | -25.508 | 0.000 | 0.012 | | 0.012 | Dimo-Simonin et al 2000 |
| Spain | SPA | 312 | 174 | 0.974 | 0.005 | -25.156 | 0.000 | 0.011 | | 0.010 | Alvarez et al 2007 |
| Timau | TIM | 46 | 17 | 0.900 | 0.026 | -3.843 | 0.097 | 0.043 | | 0.020 | This study |
| Tirana | TIR | 42 | 29 | 0.966 | 0.017 | -25.323 | 0.000 | 0.012 | | 0.020 | Bosch et al 2006 |
| Tracia | TRA | 25 | 20 | 0.963 | 0.029 | -11.901 | 0.000 | 0.014 | | 0.017 | Bosch et al 2006 |
| Udine | UDI | 51 | 32 | 0.903 | 0.038 | -25.641 | 0.000 | 0.012 | | 0.025 | Brisighelli et al 2012 |
| Val Badia (Ladins) | LVB | 56 | 28 | 0.945 | 0.017 | -18.660 | 0.000 | 0.018 | | 0.030 | Thomas et al 2008 |
| Val d'Adige | TVA | 56 | 42 | 0.988 | 0.006 | -25.576 | 0.000 | 0.012 | | 0.013 | Coia et al 2012 |
| Val d'Isarco | VIS | 34 | 19 | 0.961 | 0.015 | -9.411 | 0.000 | 0.045 | | 0.030 | Pichler et al 2006 |
| Val di Fassa (Ladins) | LVF | 47 | 25 | 0.932 | 0.026 | -14.141 | 0.000 | 0.017 | | 0.013 | Coia et al 2012 |
| Val Gardena (Ladins) | LVG | 46 | 18 | 0.867 | 0.036 | -6.072 | 0.016 | 0.035 | | 0.035 | Thomas et al 2008 |
| Val Pusteria | VPU | 37 | 14 | 0.890 | 0.032 | -3.831 | 0.050 | 0.031 | | 0.061 | Pichler et al 2006 |
| Val Venosta | VAV | 112 | 49 | 0.942 | 0.014 | -25.888 | 0.000 | 0.013 | | 0.012 | Pichler et al 2006 |
| Vojvodina | VOJ | 104 | 68 | 0.981 | 0.006 | -25.744 | 0.000 | 0.010 | | 0.015 | Zgonjanin et al 2010 |
| West Austria | AUW | 101 | 66 | 0.950 | 0.017 | -25.719 | 0.000 | 0.008 | | 0.008 | Parson et al 1998 |
| West Germany | GRW | 50 | 34 | 0.941 | 0.027 | -25.889 | 0.000 | 0.011 | | 0.018 | Baasner et al 1998 |
| West Slovakia | SLW | 70 | 52 | 0.979 | 0.010 | -25.580 | 0.000 | 0.011 | | 0.009 | Malyarchuk et al 2008 |

Abbreviations: Acr, Acronym; n, Sample size; k, Number of haplotypes; HD, Haplotype Diversity; Avg Fst, Average Fst; r, Harpending’s Raggedness.

*significance at 5% level (p<0.02; see Fu 1997)

**References**

Achilli A, Olivieri A, Pala M *et al.* (2007) Mitochondial DNA variation of modern Tuscans supports the Near Eastern origin of Etruscans. *American Journal of Human Genetics*, **80**, 759-768.

Alvarez JC, Johnson DLE, Lorente JA *et al.* (2007) Characterization of human control region sequences for Spanish individuals in a forensic mtDNA data set. *Legal Medicine*, **9**, 293-304.

Baasner A, Schäfer C, Junge A, Madea B (1998) Polymorphic sites in human mitochondrial DNA control region sequences: population data and maternal inheritance. *Forensic Science International*, **98**, 169-178.

Babalini C, Martínez-Labarga C, Tolk HV *et al.* (2005) The population history of the Croatian linguistic minority of Molise (southern Italy): a maternal view. *European Journal of Human Genetics*, **13**, 902-912.

Belledi M, Poloni ES, Casalotti R *et al.* (2000) Maternal and paternal lineages in Albania and the genetic structure of Indo-European populations. *European Journal of Human Genetics*, **8**, 480-486.

Bini C, Ceccardi S, Luiselli D *et al.* (2003) Different informativeness of the three hypervariable mitochondrial DNA regions in the population of Bologna (Italy). *Forensic Science International*, **135**, 48-52.

Bosch E, Calafell F, González-Neira A, Flaiz C *et al.* (2006) Paternal and maternal lineages in the Balkans show a homogeneous landscape over linguistic barriers, except for the isolated Aromuns. *Annals of Human Genetics*, **70**, 459-487.

Brandstätter A, Egyed B, Zimmermann B *et al.* (2007) Migration rates and genetic structure of two Hungarian ethnic groups in Transylvania, Romania. *Annals of Human Genetics*, **71**, 791-803.

Brisighelli F, Alvarez-Iglesias V, Fondevila M *et al.* (2012) Uniparental markers of contemporary Italian population reveals details of its pre-Roman heritage. *PloS One*, **7**: e50794.

Cardoso S, Alfonso-Sánchez MA, Valverde L *et al.* (2011) The maternal legacy of Basques in Northern Navarre: new insights into the mitochondrial DNA diversity of the Franco-Cantabrian Area. *American Journal of Physical Anthropology*, **145**, 480-488.

Coia V, Boschi I, Trombetta F *et al.* (2012) Evidence of high genetic variation among linguistically diverse populations on a micro-geographic scale: a case study of the Italian Alps. *Journal of Human Genetics*, **57**, 254-260.

Dimo-Simonin N, Grange F, Taroni F, Brandt-Casadevall C, Mangin P (2000) Forensic evaluation of mtDNA in a population from south west Switzerland. *International Journal of Legal Medicine*, **113**, 89-97.

Fu YX (1997) Statistical tests of neutrality of mutations against population growth, hitchhiking and backgroud selection. *Genetics*, **147**, 915-925.

Irwin J, Egyed B, Saunier J *et al.* (2007) Hungarian mtDNA population databases from Budapest and the Baranya county Roma. *International Journal of Legal Medicine*, **121**, 377-383.

Lutz S, Weisser HJ, Heizmann J, Pollak S (1998) Location and frequency of polymorphic positions in the mtDNA control region of individuals from Germany. *International Journal of Legal Medicine*, **111**, 67-77.

Malyarchuck BA, Grzybowski T, Derenko MV *et al.* (2003) Mitochondrial DNA variability in Bosnians and Slovenians. *Annals of Human Genetics*, **67**, 412-425.

Malyarchuk BA, Perkova MA, Derenko MV *et al.* (2008) Mitochondrial DNA variability in Slovaks, with application to the Roma Origin. *Annals of Human Genetics*, **72**, 228-240.

Parson W, Parsons TJ, Scheithauer R, Holland MM (1998) Population data for 101 Austrian Caucasian mitochondrial DNA d-loop sequences: Application of mtDNA sequence analysis to a forensic case. *International Journal of Legal Medicine*, **111**, 124-132.

Pereira L, Prata MJ, Amorim A (2000). Diversity of mtDNA lineages in Portugal: not a genetic edge of European variation. *Annals of Human Genetics*, **64**, 491-506.

Pichler I, Mueller JC, Stefanov SA *et al.* (2006) Genetic structure in contemporary South Tyrolean isolated populations revealed by analysis of Y-chromosome, mtDNA, and Alu polymorphisms. *Human Biology*, **78**, 441-464.

Poetsch M, Wittig H, Krause D, Lignitz E (2003) Mitochondrial diversity of a northeast German population sample. *Forensic Science International*, **137**, 125-132.

Richards M, Macaulay V, Hickey E *et al.* (2000) Tracing European founder lineages in the Near Eastern mtDNA pool. *American Journal of Human Genetics*, **67**, 1251-1276.

Rousselet F, Mangin P (1998) Mitochondrial DNA polymorphisms: a study of 50 French Caucasian individuals and application to forensic casework. *International Journal of Legal Medicine*, **111**, 292-298.

Tagliabracci A, Turchi C, Buscemi L, Sassaroli C (2001) Polymorphism of the mitochondrial DNA control region in Italians. *International Journal of Legal Medicine*, **114**, 224-228.

Thomas MG, Barnes I, Weale ME *et al.* (2008) New genetic evidence supports isolation and drift in the Ladin communities of the South Tyrolean Alps but not an ancient origin in the Middle East. *European Journal of Human Genetics*, **16**, 124-134.

Vanacek T, Vorel F, Sip M (2004) Mitochondrial DNA D-loop hypervariable regions: Czech population data. *International Journal of Legal Medicine*, **118**, 14-18.

Zgonjanin D, Veselinović I, Kubat M *et al.* (2010) Sequence polymorphism of the mitochondrial DNA control region in the population of Vojvodina Province, Serbia. *Legal Medicine*, **12**, 104-107.

Zimmermann B, Brandstätter A, Duftner N *et al.* (2007) Mitochondrial DNA control region population data from Macedonia. *Forensic Science International: Genetics*, **1**, e4-e9.
