# Supplementary material for: Detecting Genetic Isolation in Human Populations: A Study of European Language Minorities
Source: PLoS One. 2013 Feb 13;8(2):e56371. doi: 10.1371/journal.pone.0056371 (PMC3572090; doi:10.1371/journal.pone.0056371)
Supplement: Table S3 — Haplogroup frequency distribution in populations under study. (DOC) [file pone.0056371.s004.doc]

**Supplementary Table S3. Haplogroup frequency distribution in populations under study.**

| **Haplogroup** | **Lessinia (n=40)** | **Sappada (n=59)** | **Sauris (n=48)** | **Timau (n=46)** |
| --- | --- | --- | --- | --- |
|
| H | 5.00 | 6.78 | 2.08 | 13.04 |
| H1 | 7.50 | 3.39 | 6.25 | 8.70 |
| H1c4b | 2.50 |  |  |  |
| H11a | 2.50 |  |  |  |
| H11a1 |  |  | 2.08 |  |
| H11a2 | 2.50 |  |  |  |
| H2 | 5.00 | 1.69 | 2.08 |  |
| H3 | 5.00 |  |  | 6.52 |
| H4 |  |  |  | 6.52 |
| H5 | 10.00 | 10.17 | 16.67 | 2.17 |
| H5a | 2.50 |  |  |  |
| H5r | 2.50 |  |  |  |
| H6a | 10.00 |  |  |  |
| H66a | 2.50 |  |  |  |
| H8 | 2.50 |  |  |  |
| H20 |  |  | 6.25 |  |
| HV |  |  |  | 2.17 |
| HV0 | 2.50 |  | 2.08 | 2.17 |
| HV1a1 | 2.50 |  |  |  |
| I5a | 2.50 |  |  |  |
| J1b1a |  |  | 2.08 |  |
| J1b1a1 |  |  | 4.17 |  |
| J1c2 |  |  | 2.08 | 4.35 |
| J1c2c |  |  |  | 2.17 |
| J1d |  |  |  | 2.17 |
| K1 | 10.00 | 44.10 | 8.33 |  |
| N1e' I |  | 5.08 |  | 2.17 |
| R2'JT |  |  |  | 2.17 |
| T |  |  |  | 4.35 |
| T1a |  | 1.69 |  |  |
| T1a1'3 | 10.00 |  | 2.08 |  |
| T2 |  | 1.69 |  | 17.40 |
| T2a1a1 |  | 3.39 |  |  |
| T2b23a |  | 1.69 |  |  |
| T2f1 |  |  |  | 2.17 |
| U |  |  |  | 2.17 |
| U1a'c |  | 1.69 |  |  |
| U2e1 | 2.50 | 1.69 | 4.17 | 6.52 |
| U4 |  | 13.56 | 4.17 | 10.90 |
| U5a |  | 1.69 | 2.10 |  |
| U5a1 |  |  |  | 2.17 |
| U5a1b1 |  |  | 4.17 |  |
| U5a2 | 2.50 |  | 2.08 |  |
| U5a2a1b1 |  |  | 18.75 |  |
| U5b | 2.50 |  |  |  |
| V | 5.00 |  |  |  |
| W |  | 1.70 | 6.25 |  |
| X |  |  | 2.08 |  |
|  |  |  |  |  |
