# Supplementary material for: Detecting Genetic Isolation in Human Populations: A Study of European Language Minorities
Source: PLoS One. 2013 Feb 13;8(2):e56371. doi: 10.1371/journal.pone.0056371 (PMC3572090; doi:10.1371/journal.pone.0056371)
Supplement: Table S4 — Estimates of effective population size, gene flow and splitting time for all runs performed. (DOC) [file pone.0056371.s005.doc]

**Supplementary Table S4. Estimates of effective population size, gene flow and splitting time for all runs performed.**

| **Population pair** | **Run** | **N1** | **N2** | **NA** | **m 1-2** | **m 2-1** | **t** |
| --- | --- | --- | --- | --- | --- | --- | --- |
| **Sappada vs Cadore** | 1 | 5767 (3460-17300) | 86500 (49593-515541) | 3460 (1153-19607) | 0.74 (0.05-34.22) | 0.35 (0.07-2.96) | 38944 (22913-50997) |
|  | 2 | 5767 (3460-17300) | 86500 (49593-439421) | 3460 (1153-19607) | 0.70 (0.05-28.25) | 0.35 (0.07-2.95) | 39156 (23586-50997) |
|  | 3 | 5767 (3460-17300) | 86500 (49593-444034) | 3460 (1153-19607) | 0.70 (0.05-29.29) | 0.36 (0.07-2.97) | 38118 (23739-50939) |
|  | Average | 5767 (3460-17300) | 86500 (49593-466332) | 3460 (1153-19607) | 0.71 (0.05-30.59) | 0.35 (0.07-2.96) | 38739 (23413-50978) |
|  |  |  |  |  |  |  |  |
| **Sauris vs Udine** | 1 | 17300 (8073-40367) | 93420 (56513-1161409) | 5767 (3460-21913) | 0.84 (0.06-77.10) | 0.40 (0.02-5.22) | 22605 (13417-36465) |
|  | 2 | 17300 (8073-40367) | 93420 (54207-1009169) | 5767 (3460-19607) | 0.84 (0.06-66.15) | 0.45 (0.02-5.43) | 22240 (13571-37022) |
|  | 3 | 17300 (8073-40367) | 91113 (56513-1073756) | 5767 (3460-19607) | 0.78 (0.06-70.83) | 0.44 (0.02-5.28) | 22663 (13623-37349) |
|  | Average | 17300 (8073-40367) | 92651 (55744-1081445) | 5767 (3460-20376) | 0.82 (0.06-71.36) | 0.43 (0.02-5.31) | 22502 (13537-36945) |
|  |  |  |  |  |  |  |  |
| **Timau vs Udine** | 1 | 17300 (8073-44980) | 114180 (68047-1936451) | 8073 (1153-26527) | 1.26 (0.10-247.01) | 0.41 (0.02-5.87) | 19434 (11130-48190) |
|  | 2 | 17300 (8073-42673) | 118794 (65740-1917998) | 8073 (1153-26527) | 1.01 (0.10-228.70) | 0.41 (0.02-5.62) | 19022 (10718-48440) |
|  | 3 | 17300 (8073-44980) | 121100 (68047-1941064) | 8073 (1153-26527) | 1.39 (0.10-237.91) | 0.40 (0.02-5.83) | 18815 (10770-47517) |
|  | Average | 17300 (8073-44211) | 118025 (67278-1931834) | 8073 (1153-26527) | 1.22 (0.10-237.87) | 0.41 (0.02-5.77) | 19090 (10873-48049) |
|  |  |  |  |  |  |  |  |
| **Sappada vs C-W Europe** | 1 | 5767 (3460-12687) | 358687 (198374-1936451) | 8073 (3460-24220) | 23.21 (4.33-205.91) | 0.42 (0.10-2.21) | 36829 (24777-49901) |
|  | 2 | 5767 (3460-12687) | 360994 (213367-1938758) | 8073 (3460-24220) | 23.66 (4.75-208.58) | 0.42 (0.10-2.16) | 35638 (24470-50266) |
|  | 3 | 5767 (3460-12687) | 370221 (217980-1959518) | 8073 (3460-24220) | 24.27 (5.03-207.55) | 0.42 (0.10-2.19) | 35522 (24374-50209) |
|  | Average | 5767 (3460-12687) | 363301 (209907-1944909) | 8073 (3460-24220) | 23.71 (4.70-207.35) | 0.42 (0.10-2.19) | 35996 (24540-50125) |
|  |  |  |  |  |  |  |  |
| **Sauris vs C-W Europe** | 1 | 17300 (8073-38060) | 626261 (314861-2229398) | 5767 (1153-19607) | 60.08 (14.22-342.83) | 0.41 (0.02-4.27) | 36618 (22605-50208) |
|  | 2 | 17300 (8073-38060) | 570901 (301021-2229398) | 5767 (1153-19607) | 55.00 (14.09-341.91) | 0.33 (0.02-4.46) | 37445 (23432-50170) |
|  | 3 | 17300 (8073-35753) | 589354 (294101-2227092) | 5767 (1153-19607) | 56.54 (11.81-337.85) | 0.38 (0.02-3.91) | 36830 (22394-49997) |
|  | Average | 17300 (8073-37291) | 595505 (303328-2228629) | 5767 (1153-19607) | 57.21 (13.37-340.36) | 0.37 (0.02-4.21) | 36964 (22810-50125) |
|  |  |  |  |  |  |  |  |
| **Timau vs C-W Europe** | 1 | 14993 (5767-42673) | 236433 (162620-1821117) | 8073 (3460-26527) | 1.72 (0.24-209.56) | 0.55 (0.03-5.90) | 28948 (19434-49440) |
|  | 2 | 14993 (5767-44980) | 238741 (162620-1828038) | 8073 (3460-28833) | 1.34 (0.24-204.27) | 0.56 (0.02-6.28) | 29814 (18857-49017) |
|  | 3 | 14993 (5767-42673) | 243354 (164927-1864944) | 10380 (3460-28833) | 1.77 (0.24-206.85) | 0.61 (0.03-6.14) | 31582 (18703-48805) |
|  | Average | 14993 (5767-43442) | 239509 (163389-1838033) | 8842 (3460-28064) | 1.61 (0.24-206.89) | 0.57 (0.03-6.11) | 30115 (18998-49087) |

Abbreviations: N1, N2 and NA, effective population size population 1, population 2 and ancestral population; m1-2, effective number of haplotypes migrating from population 1 to population 2, per year; m2-1, effective number of haplotypes migrating from population 2 to population 1, per year; t, splitting time in years. 95% credibility intervals in parentheses.
