# Supplementary material for: Detecting Genetic Isolation in Human Populations: A Study of European Language Minorities
Source: PLoS One. 2013 Feb 13;8(2):e56371. doi: 10.1371/journal.pone.0056371 (PMC3572090; doi:10.1371/journal.pone.0056371)
Supplement: Table S5 — Measures of MCMC mixing behavior. (DOC) [file pone.0056371.s006.doc]

**Supplementary Table S5. Measures of MCMC mixing behavior.**

|  | | **Autocorrelation Estimates (1 * 106 steps)** | | | **Effective Sample Size Estimates** | | | **Marginal Peak locations** | | | | |
| --- | --- | --- | --- | --- | --- | --- | --- | --- | --- | --- | --- | --- |
|  |  |  |  |  |  |  |  |  |  |  |  |  |
| **Population pair** | **Run** | **Log[P]** | **t** | **tmrca** | **Log[P]** | **t** | **tmrca** | **q1 SET0-SET1** | **q2 SET0-SET1** | **qa SET0-SET1** | **m1>2 SET0-SET1** | **m2>1 SET0-SET1** |
| **Sappada vs Cadore** | 1 | 0.0264 | 0.0202 | 0.0027 | 161475 | 43524 | 152461 | 17.468 – 17.397 | 223.891 – 224.512 | 7.898 – 9.072 | 0.585 – 0.592 | 0.083 – 0.084 |
|  | 2 | 0.0312 | 0.0452 | -0.0546 | 129168 | 207022 | 160244 | 17.288 – 17.402 | 226.962 – 225.145 | 7.993 – 8.851 | 0.592 – 0.589 | 0.083 – 0.075 |
|  | 3 | 0.0299 | 0.0012 | -0.0234 | 133 | 230484 | 68492 | 17.476 – 17.377 | 227.070 – 227.348 | 9.593 – 9.499 | 0.588 – 0.612 | 0.080 – 0.074 |
|  |  |  |  |  |  |  |  |  |  |  |  |  |
| **Sauris vs Udine** | 1 | -0.0013 | -0.0014 | -0.0098 | 140636 | 79513 | 113424 | 45.780 – 45.568 | 239.344 – 241.031 | 17.822 – 17.350 | 0.216 – 0.231 | 0.087 – 0.089 |
|  | 2 | 0.0032 | -0.0648 | -0.0028 | 117676 | 67428 | 141419 | 45.587 – 45.582 | 239.920 – 241.848 | 16.757 – 17.460 | 0.236 – 0.268 | 0.084 – 0.091 |
|  | 3 | -0.0096 | 0.0048 | 0.0509 | 66977 | 56397 | 151812 | 45.536 – 45.697 | 242.110 – 236.838 | 17.503 – 17.490 | 0.252 – 0.242 | 0.088 – 0.078 |
|  |  |  |  |  |  |  |  |  |  |  |  |  |
| **Timau vs Udine** | 1 | 0.0386 | 0.0575 | 0.0416 | 76363 | 133207 | 125326 | 43.335 – 41.394 | 309.129 – 288.307 | 22.685 – 19.934 | 0.186 – 0.262 | 0.074 – 0.127 |
|  | 2 | 0.0448 | 0.0713 | 0.0595 | 163480 | 65774 | 50414 | 42.534 – 42.473 | 322.577 – 294.827 | 23.537 – 20.683 | 0.226 – 0.239 | 0.077 – 0.089 |
|  | 3 | 0.0374 | 0.0247 | -0.0168 | 61281 | 128484 | 147215 | 42.710 – 44.704 | 311.354 – 314.125 | 23.045 – 24.469 | 0.224 – 0.219 | 0.125 – 0.095 |
|  |  |  |  |  |  |  |  |  |  |  |  |  |
| **Sappada vs C-W Europe** | 1 | -0.0584 | 0.0016 | 0.0111 | 36714 | 68000 | 122315 | 12.033 – 13.284 | 934.686 – 933.884 | 21.469 – 21.739 | 0.693 – 0.722 | 0.627 – 0.615 |
|  | 2 | 0.0377 | -0.0168 | 0.0482 | 75210 | 126998 | 108286 | 12.726 – 13.190 | 910.598 – 963.111 | 21.270 – 21.953 | 0.717 – 0.695 | 0.623 – 0.640 |
|  | 3 | -0.0075 | 0.0337 | 0.0087 | 54648 | 61797 | 132395 | 12.920 – 12.841 | 978.059 – 942.856 | 20.051 – 22.054 | 0.712 – 0.687 | 0.640 – 0.621 |
|  |  |  |  |  |  |  |  |  |  |  |  |  |
| **Sauris vs C-W Europe** | 1 | -0.0382 | -0.0152 | -0.0088 | 76203 | 80625 | 150558 | 44.164 – 45.980 | 1613.280 – 1645.044 | 12.381 – 12.835 | 0.221 – 0.237 | 0.929 – 0.915 |
|  | 2 | -0.011 | -0.0067 | 0.0335 | 26065 | 124091 | 138975 | 46.302 – 47.019 | 1512.824 – 1458.150 | 12.576 – 13.006 | 0.191 – 0.175 | 0.935 – 0.912 |
|  | 3 | 0.0135 | -0.0113 | 0.0635 | 44125 | 131038 | 52494 | 45.457 – 43.166 | 1549.401 – 1519.534 | 13.002 – 12.342 | 0.216 – 0.208 | 0.935 – 0.911 |
|  |  |  |  |  |  |  |  |  |  |  |  |  |
| **Timau vs C-W Europe** | 1 | 0.0328 | 0.0118 | 0.0141 | 165195 | 145513 | 106190 | 36.195 – 37.037 | 630.869 – 599.935 | 24.372 – 21.297 | 0.348 – 0.357 | 0.073 – 0.064 |
|  | 2 | -0.0368 | 0.0435 | -0.0251 | 72590 | 81776 | 120859 | 38.918 – 38.519 | 619.631 – 625.032 | 22.411 – 21.932 | 0.350 – 0.368 | 0.044 – 0.068 |
|  | 3 | 0.0126 | 0.0804 | 0.0345 | 159635 | 64611 | 106663 | 39.121 – 38.047 | 627.084 – 639.327 | 25.589 – 23.471 | 0.404 – 0.373 | 0.075 – 0.063 |
|  |  |  |  |  |  |  |  |  |  |  |  |  |

Abbreviations: Log[P] = Log(P(Data|Genealogy)) + Log(P(Genealogy|Parameters)); t, splitting time; tmrca, time of the most recent common ancestor; q1, q2 and qa, effective size parameters population 1, population 2 and ancestral population; m1>2, gene flow rate per haplotype from population 2 to population 1; m2>1, gen flow rate per haplotype from population 1 to population 2.
