# Supplementary material for: Detecting Genetic Isolation in Human Populations: A Study of European Language Minorities
Source: PLoS One. 2013 Feb 13;8(2):e56371. doi: 10.1371/journal.pone.0056371 (PMC3572090; doi:10.1371/journal.pone.0056371)
Supplement: Table S6 — List of populations used for the analysis of Y chromosome STRs. (DOC) [file pone.0056371.s007.doc]

**Supplementary Table S6. List of populations used for the analysis of Y chromosome STRs.**

| **Population** | **Acr** | **n** | **References** |
| --- | --- | --- | --- |
| Andon Poci (Aromuns) | AAP | 199 | Bosch et al. 2006 |
| Dukasi (Aromuns) | ADU | 39 | Bosch et al. 2006 |
| Krusevo (Aromuns) | AKR | 43 | Bosch et al. 2006 |
| Stip (Aromuns) | AST | 58 | Bosch et al. 2006 |
| **Sappada** | **SAP** | **36** | **This study** |
| **Sauris** | **SAU** | **29** | **This study** |
| **Timau** | **TIM** | **23** | **This study** |
| Val Badia (Ladins) | LVB | 56 | Thomas et al. 2008 |
| Val di Fassa (Ladins) | LVF | 47 | Coia p.c. |
| Val Gardena (Ladins) | LVG | 46 | Thomas et al. 2008 |

**References**

Bosch E, Calafell F, González-Neira A, Flaiz C *et al.* (2006) Paternal and maternal lineages in the Balkans show a homogeneous landscape over linguistic barriers, except for the isolated Aromuns. *Annals of Human Genetics*, **70**, 459-487.

Thomas MG, Barnes I, Weale ME *et al.* (2008) New genetic evidence supports isolation and drift in the Ladin communities of the South Tyrolean Alps but not an ancient origin in the Middle East. *European Journal of Human Genetics*, **16**, 124-134.
