# Supplementary material for: Detecting Genetic Isolation in Human Populations: A Study of European Language Minorities
Source: PLoS One. 2013 Feb 13;8(2):e56371. doi: 10.1371/journal.pone.0056371 (PMC3572090; doi:10.1371/journal.pone.0056371)
Supplement: Figure S1 — Posterior distributions of parameter estimates. Parameter abbreviations: q1, q2 and qa (effective population size of populations 1,2 and ancestral), T (splitting time), m1>2 (gene flow rate per haplotype from populations 2 to 1) and m2>1 (gene flow rate per haplotype from populations 1 to 2). A) Sappada vs Cadore; B) Sappada vs Central-Western Europe; C) Sauris vs Udine; D) Sauris vs Central-Western Europe; E) Timau vs Udine; F) Timau vs Central-Western Europe. (PDF) [file pone.0056371.s008.pdf]

Supplementary Figure S1. Posterior distributions of parameter estimates of **q1**, **q2** and **qa** (effective population size of populations 1,2 and ancestral), **T** (splitting time), **m1>2** (gene flow rate per haplotype from populations 2 to 1) and **m2>1** (gene flow rate per haplotype from populations 1 to 2).

A) Sappada vs Cadore; B) Sappada vs Central-Western Europe; C) Sauris vs Udine; D) Sauris vs Central-Western Europe; E) Timau vs Udine; F) Timau vs Central-Western Europe.

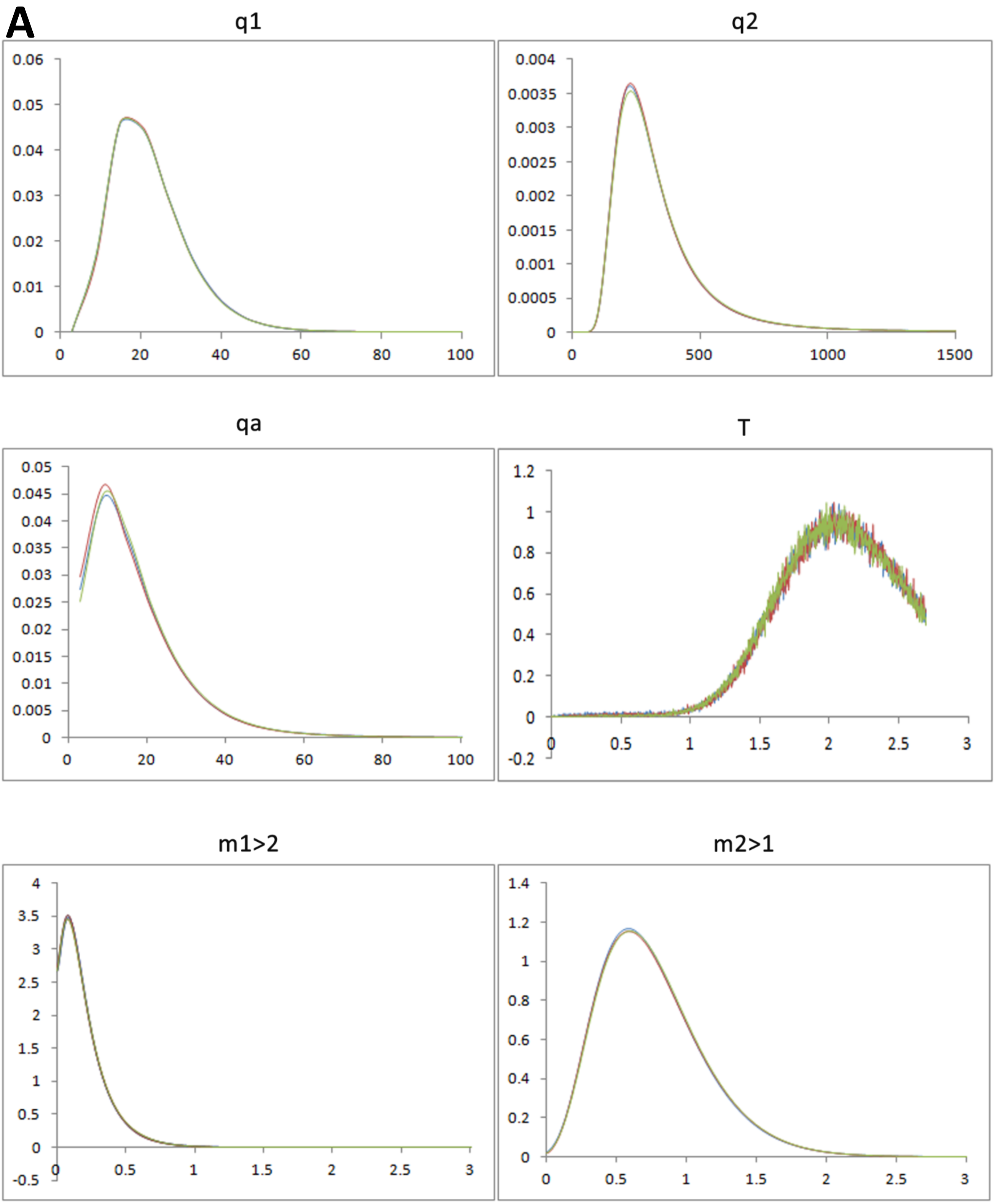

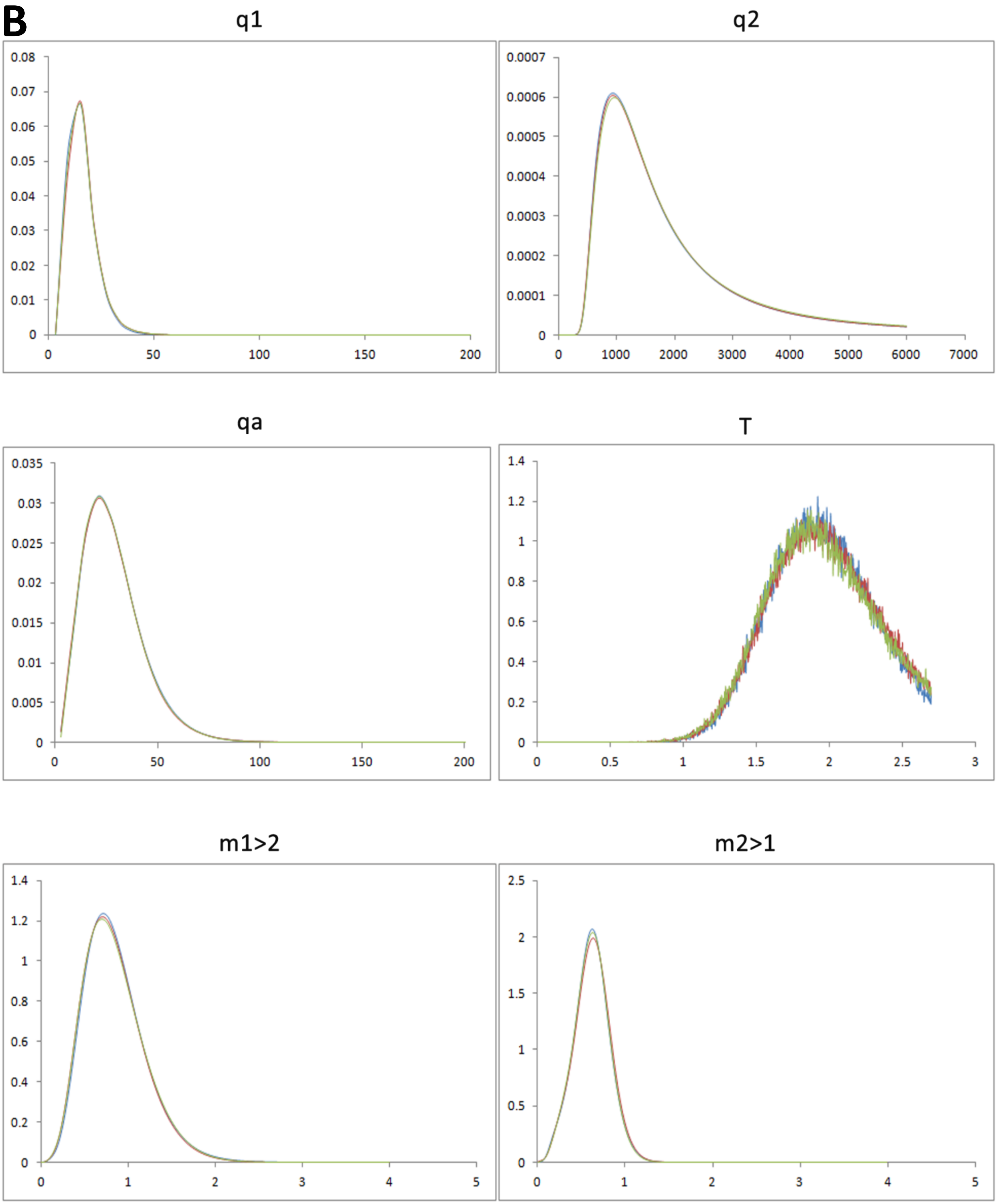
B
q1
0.08
0.07
0.06
0.05
0.04
0.03
0.02
0.01
0
0
50
100
150
200
q2
0.0007
0.0006
0.0005
0.0004
0.0003
0.0002
0.0001
0
0
1000
2000
3000
4000
5000
6000
7000
qa
0.035
0.03
0.025
0.02
0.015
0.01
0.005
0
0
50
100
150
200
T
1.4
1.2
1
0.8
0.6
0.4
0.2
0
0
0.5
1
1.5
2
2.5
3
m1>2
1.4
1.2
1
0.8
0.6
0.4
0.2
0
0
1
2
3
4
5
m2>1
2.5
2
1.5
1
0.5
0
0
1
2
3
4
5

C

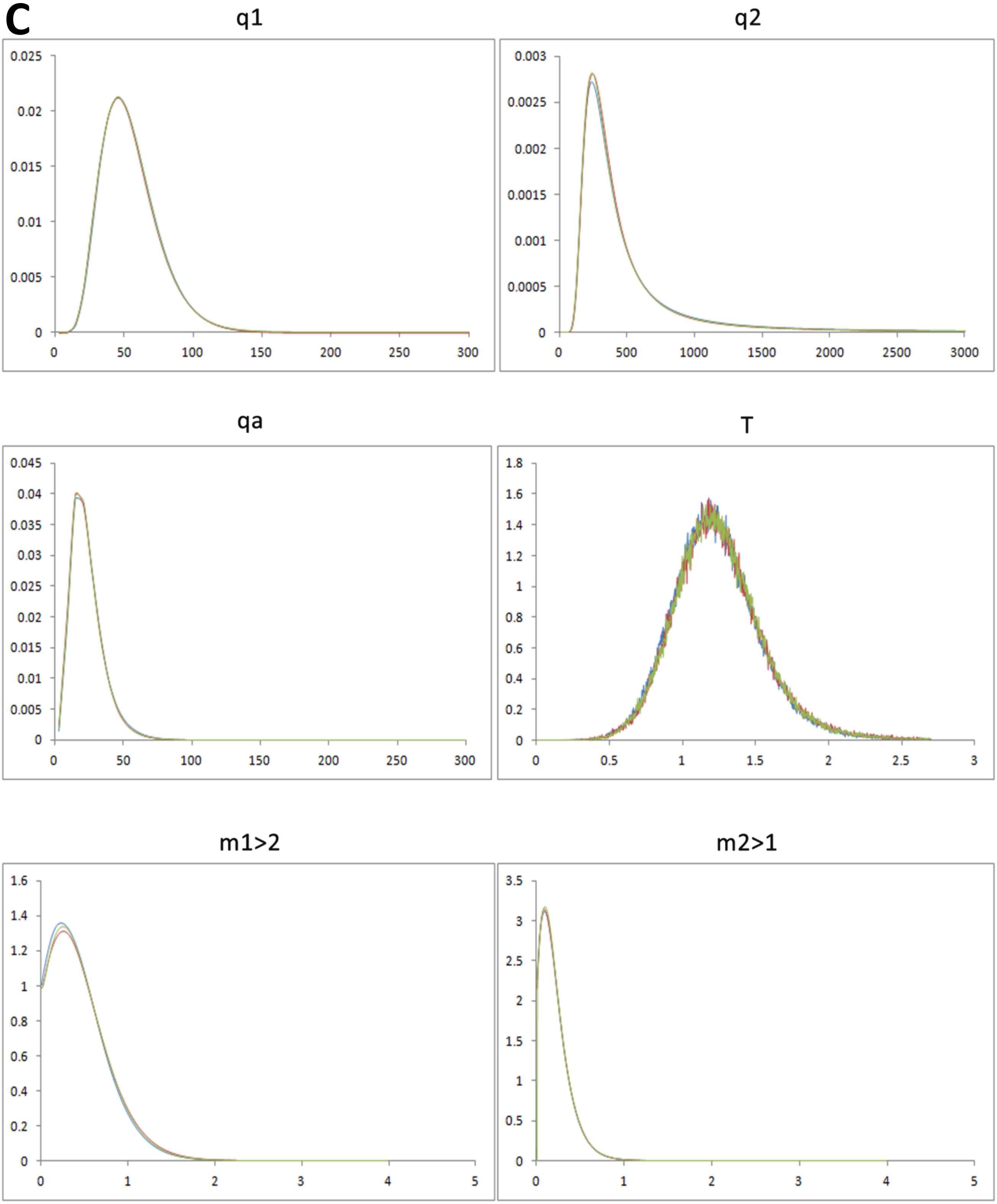
q1
0.025
0.02
0.015
0.01
0.005
0
0 50 100 150 200 250 300
q2
0.003
0.0025
0.002
0.0015
0.001
0.0005
0
0 500 1000 1500 2000 2500 3000
qa
0.045
0.04
0.035
0.03
0.025
0.02
0.015
0.01
0.005
0
0 50 100 150 200 250 300
T
1.8
1.6
1.4
1.2
1
0.8
0.6
0.4
0.2
0
0 0.5 1 1.5 2 2.5 3
m1>2
1.6
1.4
1.2
1
0.8
0.6
0.4
0.2
0
0 1 2 3 4 5
m2>1
3.5
3
2.5
2
1.5
1
0.5
0
0 1 2 3 4 5

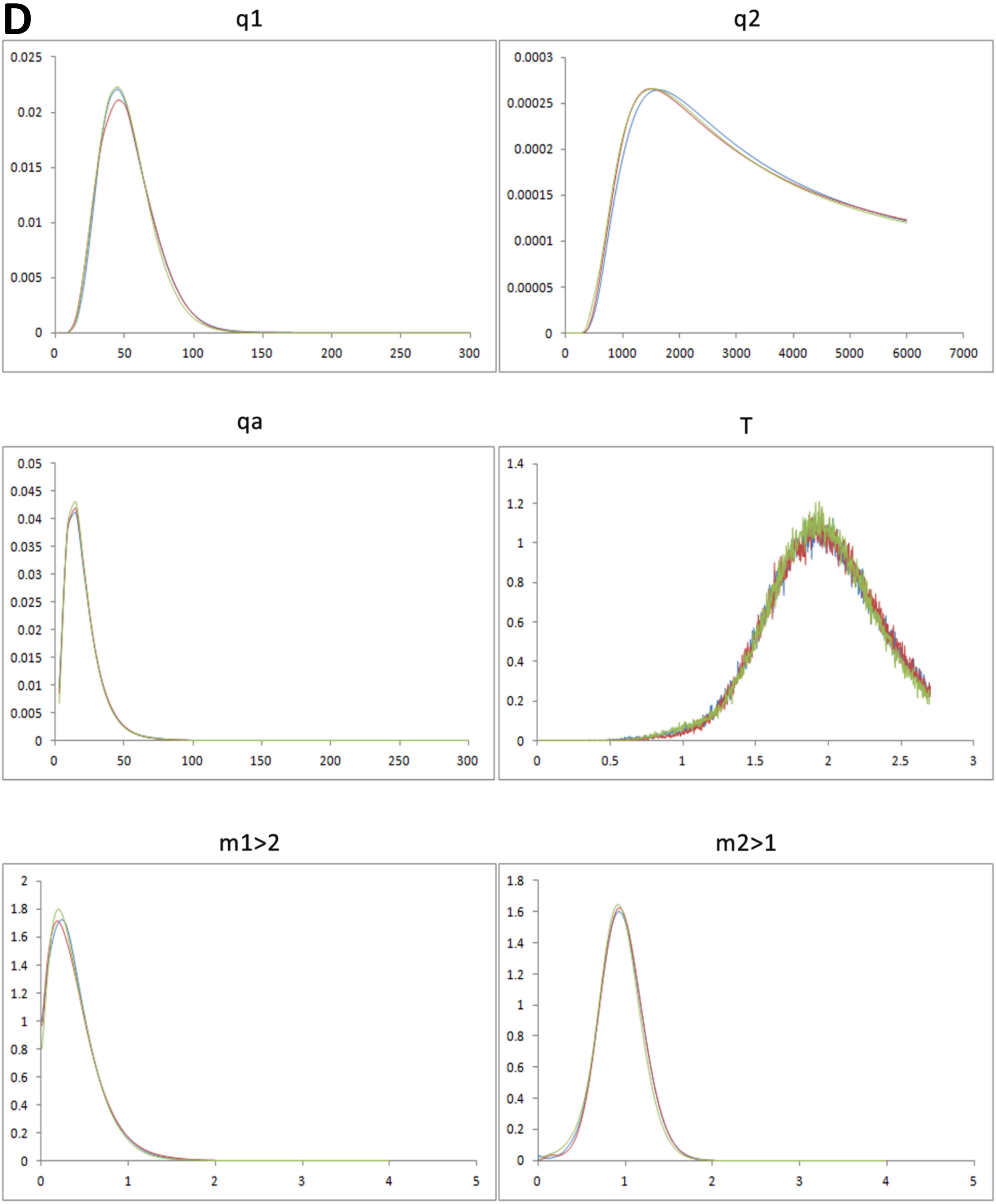
D
q1
0.025
0.02
0.015
0.01
0.005
0
0
50
100
150
200
250
300
q2
0.0003
0.00025
0.0002
0.00015
0.0001
0.00005
0
0
1000
2000
3000
4000
5000
6000
7000
qa
0.05
0.045
0.04
0.035
0.03
0.025
0.02
0.015
0.01
0.005
0
0
50
100
150
200
250
300
T
1.4
1.2
1
0.8
0.6
0.4
0.2
0
0
0.5
1
1.5
2
2.5
3
m1>2
2
1.8
1.6
1.4
1.2
1
0.8
0.6
0.4
0.2
0
0
1
2
3
4
5
m2>1
1.8
1.6
1.4
1.2
1
0.8
0.6
0.4
0.2
0
0
1
2
3
4
5

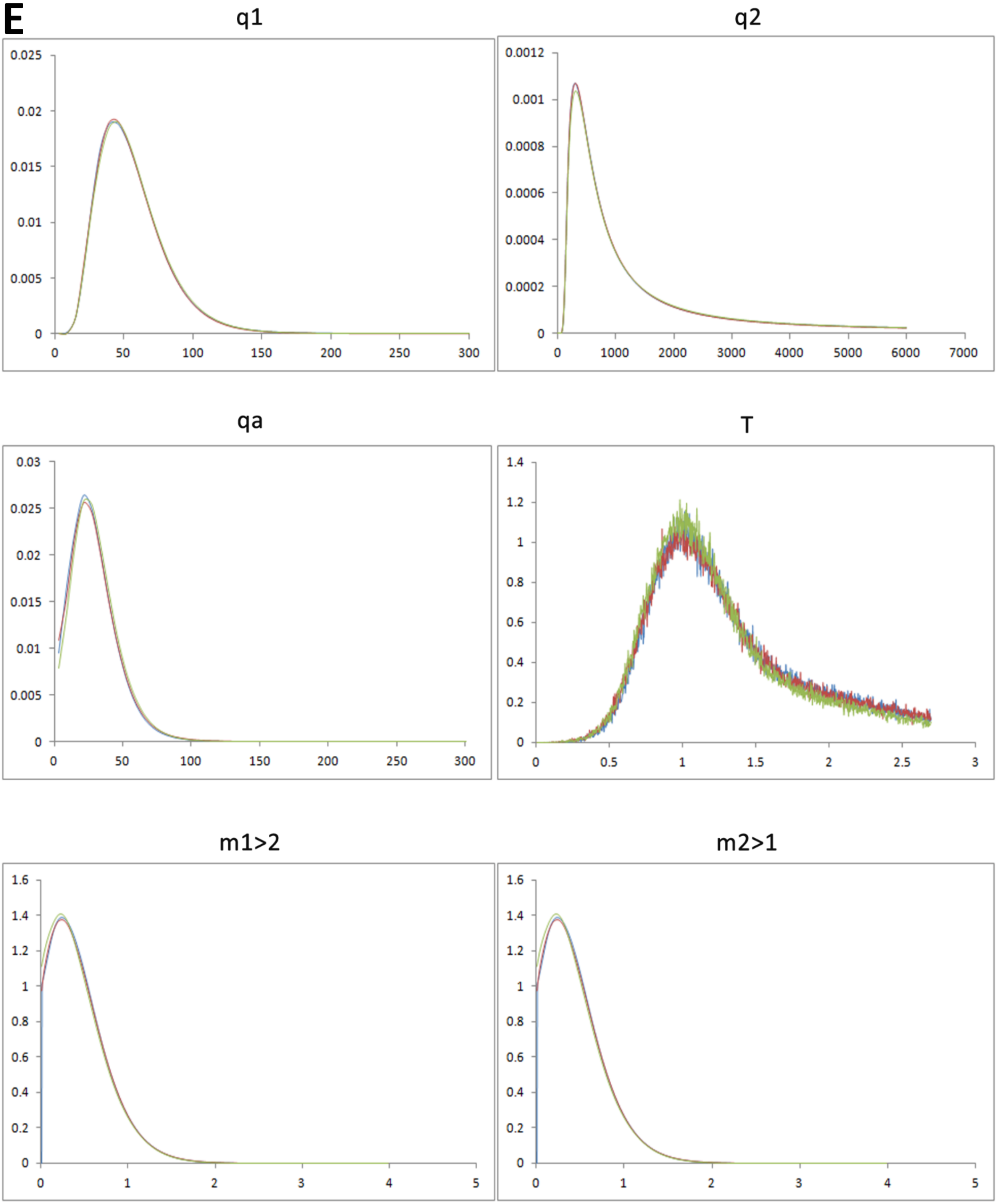
E
q1
0.025
0.02
0.015
0.01
0.005
0
0 50 100 150 200 250 300
q2
0.0012
0.001
0.0008
0.0006
0.0004
0.0002
0
0 1000 2000 3000 4000 5000 6000 7000
qa
0.03
0.025
0.02
0.015
0.01
0.005
0
0 50 100 150 200 250 300
T
1.4
1.2
1
0.8
0.6
0.4
0.2
0
0 0.5 1 1.5 2 2.5 3
m1>2
1.6
1.4
1.2
1
0.8
0.6
0.4
0.2
0
0 1 2 3 4 5
m2>1
1.6
1.4
1.2
1
0.8
0.6
0.4
0.2
0
0 1 2 3 4 5

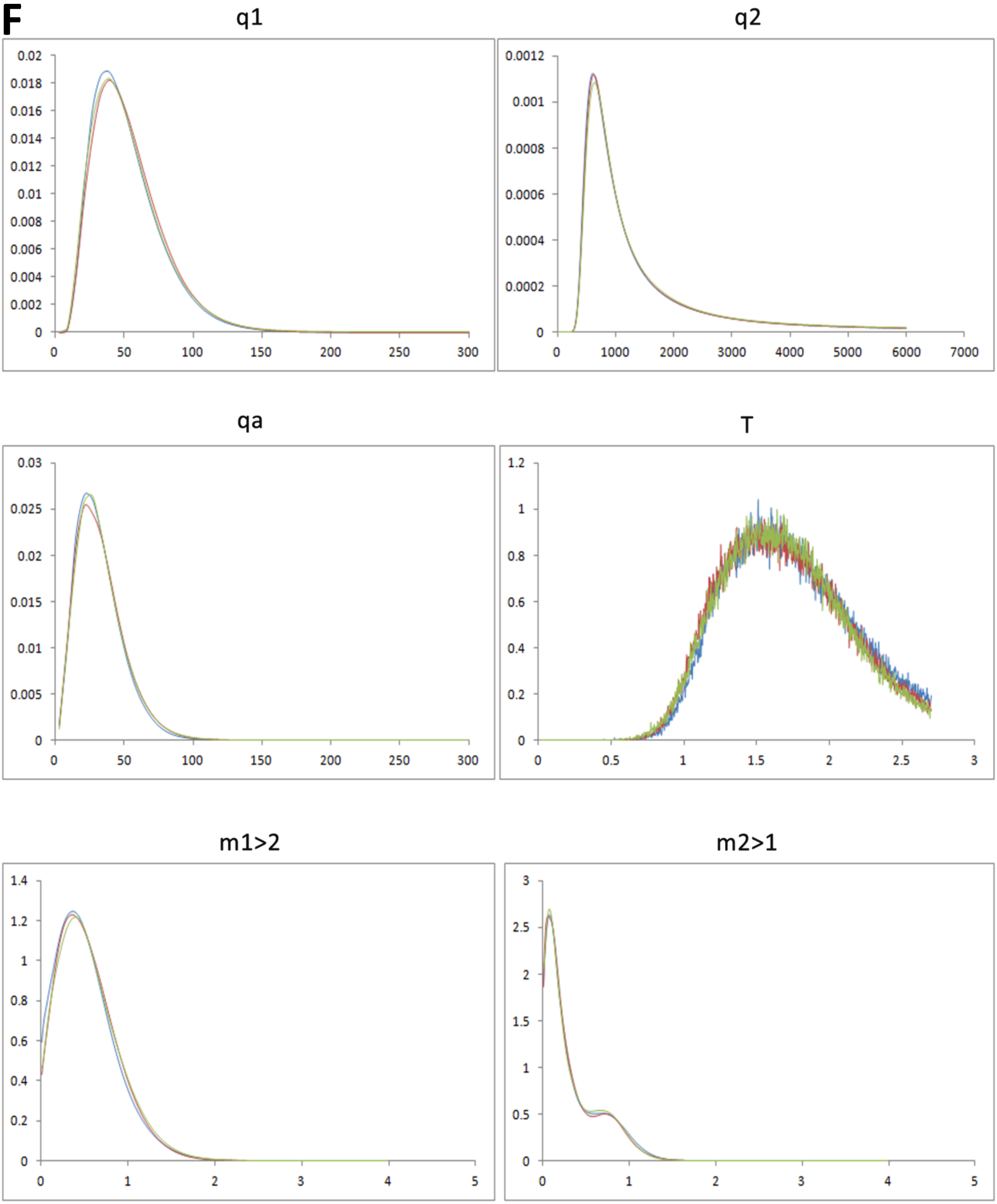

F
q1
0.02
0.018
0.016
0.014
0.012
0.01
0.008
0.006
0.004
0.002
0
0 50 100 150 200 250 300
q2
0.0012
0.001
0.0008
0.0006
0.0004
0.0002
0
0 1000 2000 3000 4000 5000 6000 7000
qa
0.03
0.025
0.02
0.015
0.01
0.005
0
0 50 100 150 200 250 300
T
1.2
1
0.8
0.6
0.4
0.2
0
0 0.5 1 1.5 2 2.5 3
m1>2
1.4
1.2
1
0.8
0.6
0.4
0.2
0
0 1 2 3 4 5
m2>1
3
2.5
2
1.5
1
0.5
0
0 1 2 3 4 5
