# Supplementary material for: Detecting Genetic Isolation in Human Populations: A Study of European Language Minorities
Source: PLoS One. 2013 Feb 13;8(2):e56371. doi: 10.1371/journal.pone.0056371 (PMC3572090; doi:10.1371/journal.pone.0056371)

Supplementary Figure S2. Evolutionary topology used for the simulations of the three scenarios.

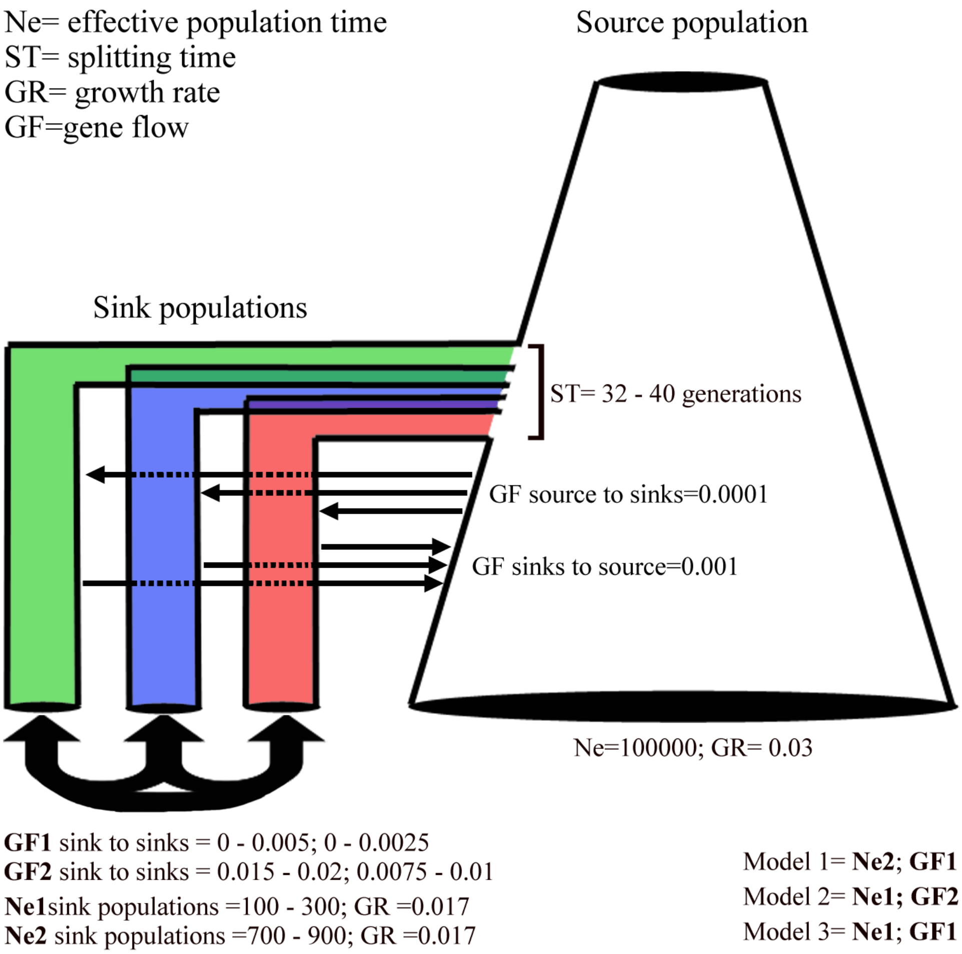

Supplement: Figure S2 — Evolutionary topology used for the simulations of the three scenarios. (PDF) [file pone.0056371.s009.pdf]
