# Supplementary material for: Detecting Genetic Isolation in Human Populations: A Study of European Language Minorities
Source: PLoS One. 2013 Feb 13;8(2):e56371. doi: 10.1371/journal.pone.0056371 (PMC3572090; doi:10.1371/journal.pone.0056371)

Supplementary Figure S3. Nucleotide pairwise mismatch distribution for the populations under study.

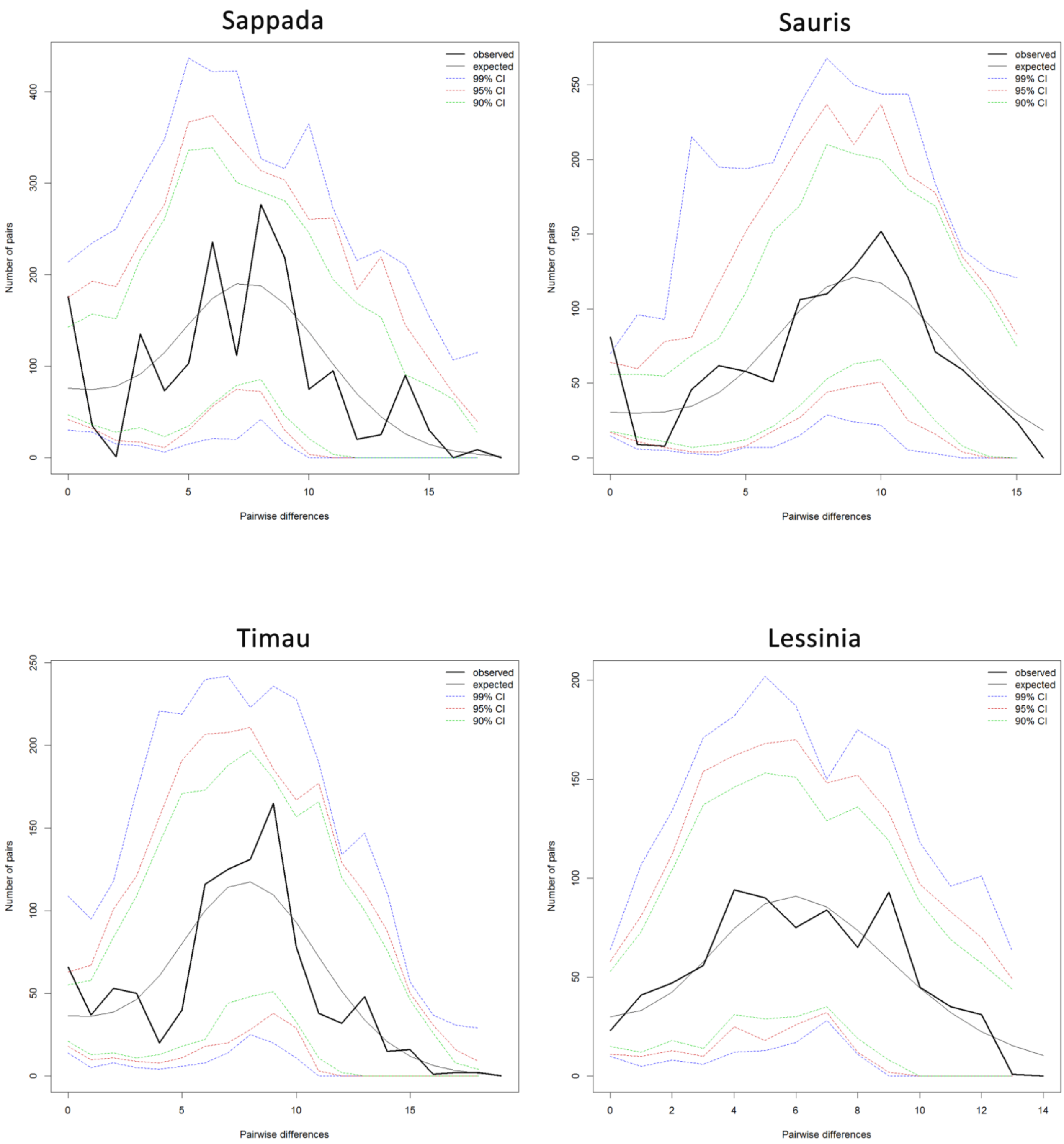

Supplement: Figure S3 — Nucleotide pairwise mismatch distribution for the populations under study. (PDF) [file pone.0056371.s010.pdf]
