# Supplementary material for: Detecting Genetic Isolation in Human Populations: A Study of European Language Minorities
Source: PLoS One. 2013 Feb 13;8(2):e56371. doi: 10.1371/journal.pone.0056371 (PMC3572090; doi:10.1371/journal.pone.0056371)

Supplementary figure S4. Fst value distributions obtained simulating a local ethnicity scenario with original (red line) and modified values (green lines) of gene flow (frames A and B) and effective size settings (frames C and D). Frames E and F show the percentual decrease of Fst modal values obtained with increasing values of gene flow and effective size.

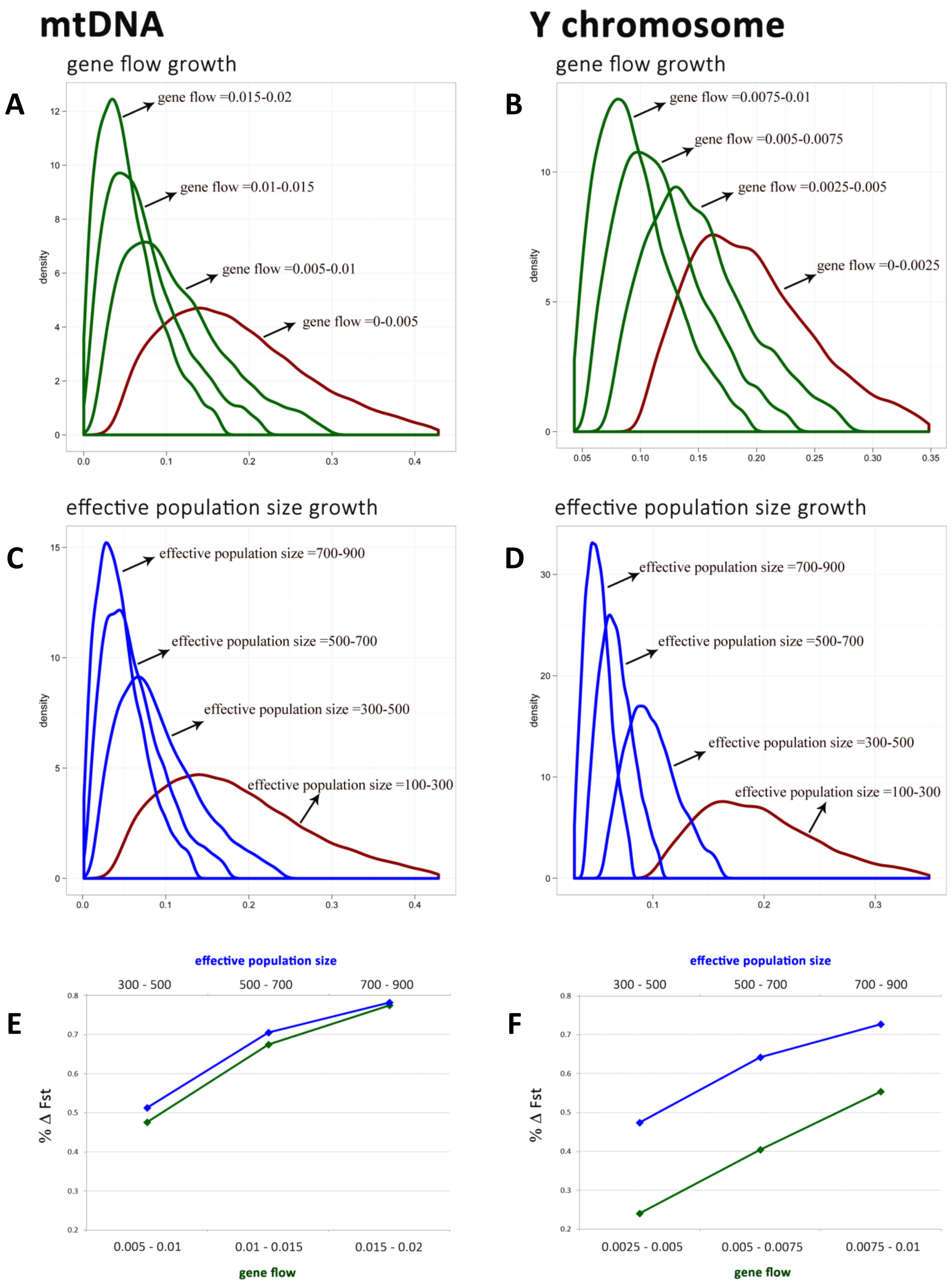

Supplement: Figure S4 — Fst value distributions of simulated scenarios. The gene flow distributions of the local ethnicity scenario with original (red line) and modified values (green lines) are shown in frames A and B whereas the effective size settings in frames C and D. Frames E and F show the percentual decrease of Fst modal values obtained with increasing values of gene flow and effective size. (PDF) [file pone.0056371.s011.pdf]
